# Inflammatory Aging‐Driven Immune Axis Imbalance and the Mechanisms of Chronicity in Elderly Atopic Dermatitis: A Systematic Review and Meta‐Analysis

**DOI:** 10.1002/iid3.70528

**Published:** 2026-09-29

**Authors:** Nan Wu, Yingjie Li, Xiaojie Wang, Yangyang Wu, Wenhao Yin

**Affiliations:** ^1^ Department of Dermatology The Affiliated Hospital of Jiaxing University, The First Hospital of Jiaxing Jiaxing China

**Keywords:** Atopic dermatitis, chronic inflammation, immune dysregulation, inflammaging, meta‐analysis, older adults with AD, Th2/Th17 axis

## Abstract

**Background:**

Elderly atopic dermatitis (elderly AD) is increasingly recognized as a chronic inflammatory skin disorder with immunopathological features that may differ from those in younger populations. Age‐related chronic low‐grade inflammation (inflammaging) may contribute to this phenotype, but its relationship with immune dysfunction and disease chronicity remains incompletely understood.

**Objective:**

To systematically review and meta‐analyze associations between inflammation‐related biomarkers and disease severity in older adults with AD and to organize the findings within a hypothesis‐generating conceptual framework of immune dysfunction.

**Methods:**

PubMed and the China National Knowledge Infrastructure (CNKI) were searched from inception through December 15, 2025, without language restrictions. Reference lists of eligible reports were also screened. Two reviewers independently screened records, extracted data, and assessed methodological quality using the Joanna Briggs Institute critical appraisal checklist for analytical cross‐sectional studies. Correlation coefficients analyses were conducted in R version 4.4.1 using the metafor package, with manual calculations in Microsoft Excel. DerSimonian‐Laird random‐effects models were used when statistical or prespecified clinical or methodological heterogeneity made a common‐effect assumption inappropriate. Otherwise, fixed‐effect models were used. The findings were also organized descriptively into four proposed pathophysiological stages.

**Results:**

Six studies met the inclusion criteria and were included for quantitative synthesis. A meta‐analysis revealed significant positive correlations between disease severity and serum total IgE, eosinophils, IL‐4, and IL‐17 levels. Among these biomarkers, eosinophil levels showed the most consistent association with disease severity across studies, whereas cytokines involved in Th2 and Th17 pathways showed considerable heterogeneity. Pathological stage‐oriented integration analysis provides a proposed conceptual model that an inflammaging‐related low‐grade inflammatory milieu may coexist with Th2‐polarized responses, aberrant Th17‐axis activation, impaired skin‐barrier repair, and AD persistence.

**Conclusion:**

Older adults with AD may exhibit multidimensional immune dysregulation beyond a simple late‐life continuation of typical AD. Our findings support a hypothesis‐generating conceptual framework in which inflammaging is associated with Th2‐skewed responses, aberrant Th17 activation, and impaired skin‐barrier repair. This framework requires validation in prospective studies but may inform future personalized therapeutic research.

## 1 Introduction

Atopic dermatitis (AD) is a chronic inflammatory skin disorder, which is characterized by impaired skin barrier function, sustained inflammation, and immune dysregulation [1, 2]. Although AD has been regarded as an allergic condition predominantly in children in previous studies, accumulating epidemiological recent evidence indicates a rising incidence of AD in older adults (age ≥ 60 years), as well as an increasing prevalence of disease persistence into later life [3, 4]. This shift is accompanied by distinct clinical phenotypes, altered treatment responses, and unique immunological profiles.

Aging is accompanied by profound immune system remodeling, encompassing both immunosenescence and inflammaging [5]. The latter refers to a chronic, low‐grade inflammatory state marked by elevated baseline levels of pro‐inflammatory cytokines and diminished immune regulatory capacity. Inflammaging is increasingly recognized as a central contributor to the pathogenesis of numerous age‐related chronic conditions [5, 6]. In elderly AD patients, while traditional Th2‐associated biomarkers (such as IgE and eosinophils) as well as various cytokines remain linked to disease activity [7], their expression patterns and clinical relevance appear to be modified. These Th2 responses generally coexist with activation of alternative pathways, including Th17 signaling. These overlapping inflammatory cascades contribute to a chronic, heterogeneous, and often treatment‐resistant disease phenotype, suggesting a more complex immunopathological landscape in aging individuals.

Despite the available clinical evidence, it remains unclear whether inflammaging merely coexists with AD in the elderly or actively contributes to disease chronicity and therapeutic resistance by fundamentally altering immune response dynamics. To address this knowledge gap, our work conducts a systematic meta‐analysis to quantitatively evaluate the association between key inflammatory biomarkers and disease severity in older adults with AD. Furthermore, we aim to organize these findings within a hypothesis‐generating framework of immune‐axis remodeling in the context of inflammaging.

## 2 Methods

### 2.1 Search Strategy

The protocol of our work was registered with the International Prospective Register of Systematic Reviews (PROSPERO) under the registration number CRD420261325988 (available at https://www.crd.york.ac.uk/prospero/). No amendments were made to the protocol after registration. The reporting of this study follows the Preferred Reporting Items for Systematic Reviews and Meta‐Analyses (PRISMA) guidelines. In detail, the scientific question was formulated using a well‐defined P–S–O framework: the population (P) consisted of elderly patients with AD, the study factor (S) encompassed inflammaging‐related biomarkers, and the outcome (O) was the clinical quantitative assessment of disease severity. And the objective of this study was to systematically synthesize extant literature to evaluate the correlation between inflammaging‐related biomarker levels and disease severity in the elderly AD population.

A comprehensive systematic search was performed across PubMed and the China National Knowledge Infrastructure (CNKI) from inception through December 15, 2025. No language restrictions were applied, and reference lists of eligible reports were manually screened. In PubMed, a strategy combining Medical Subject Headings (MeSH) terms and free‐text keywords was utilized. Search terms included:

1. Disease‐related: “Dermatitis, Atopic,” “Atopic Dermatitis,” and “Eczema.”
2. Population‐related: “Aged,” “Aging,” “Geriatric,” “Elderly,” and “older adult.”
3. Biomarker‐related: “Inflammation,” “Biomarkers,” “Cytokines,” and “Chemokines.”
4. Outcome‐related: “Severity of Illness Index,” “Disease Progression,” “Eczema Area and Severity Index (EASI),” and “Scoring Atopic Dermatitis (SCORAD)”.

These terms were integrated using Boolean operators (and/or) to maximize retrieval sensitivity.

For the Chinese CNKI database, index terms such as “atopic dermatitis” and “elderly” were used, supplemented by full‐text searches for “EASI,” “SCORAD,” “scoring”, and “severity” in Chinese, thereby capturing data from the Chinese population on the clinical characteristics and inflammatory profiles of elderly AD populations. Furthermore, reference lists of all identified articles were manually screened to minimize potential omissions. Complete database‐specific search strings for PubMed and CNKI are provided in the Supplementary File.

### 2.2 Inclusion and Exclusion Criteria

The literature screening process was conducted in two phases: title and abstract screening (Round 1), followed by full‐text screening (Round 2). In the initial screening stage, the predefined P–S–O criteria were strictly followed, and the literature was rapidly assessed based on titles and abstracts. Eligible studies were required to include elderly AD patients, typically defined as individuals aged 60 years or older, while studies involving children, adolescents, or adult populations without age stratification were excluded. The biomarker‐related factors had to measure inflammatory or inflammaging‐related biomarkers in blood or skin samples, such as cytokines, chemokines, eosinophils, or IgE. Studies limited to genetic characteristics and microbiome analyses, or lacking assessment of inflammatory parameters, were excluded. For study outcomes, eligible studies were required to use AD disease severity assessment tools, such as EASI or SCORAD index, whereas studies that reported clinical diagnoses without quantitative severity assessment were excluded. Additionally, only original studies, including cross‐sectional studies, cohort studies, case‐control studies, or clinical studies, were included, while reviews, meta‐analyses, case reports, editorials, and studies based on animal or in vitro models were excluded.

During the initial screening phase, each article was categorized as “Include,” “Exclude,” or “Uncertain.” Articles categorized as “uncertain” due to insufficient information in the abstract were advanced to full‐text review. In the full‐text review stage, papers were evaluated in detail to confirm eligibility based on participant age, biomarker measurement methods, sample sources, disease severity assessment tools, and the availability of correlation coefficients or sufficient data to calculate them. Only studies satisfying all inclusion criteria were ultimately included in the quantitative synthesis.

### 2.3 Methodological Quality Assessment

Two reviewers independently assessed the methodological quality of the included studies using the Joanna Briggs Institute Critical Appraisal Checklist for Analytical Cross‐Sectional Studies, which evaluates eight domains including appropriateness of the sampling frame, adequacy of the sampling method, sample size justification, validity and reliability of exposure and outcome measurements, identification and handling of confounding factors, appropriateness of statistical analysis, and adequacy of response rate. Disagreements were resolved through discussion, or by consultation with a third reviewer when consensus could not be reached.

Of note, one included study (Jia et al., 2023) used squamous cell carcinoma antigen (SCCA) expression level as a laboratory surrogate of disease activity rather than a validated clinical severity instrument such as SCORAD or EASI. SCCA is a marker reflecting keratinocyte proliferation and epithelial inflammation. Although it is not a validated clinical severity score, we retained this study in the primary exploratory synthesis because it provided a quantitative, extractable biomarker‐disease activity association. We explicitly distinguish this laboratory‐based surrogate from the clinical severity scores used in the other included studies and performed a sensitivity analysis excluding this dataset to assess its influence on the overall findings.

### 2.4 Data Extraction and Statistical Analysis

Data extraction was performed independently by paired reviewers, extracting information including author, publication year, study design, sample size (*n*), age range of the study population (age ≥ 60 years), biomarker names, sample types, disease severity assessment tools, and relevant statistical indicators (correlation coefficient r and *p*‐values). Any discrepancies during the extraction process were resolved through discussion.

In statistical analysis, the original Pearson or Spearman correlation coefficients (r) were transformed to Fisher's z values, with sampling variance Vz = 1/(*n* − 3) [8]. The combined Zr and its 95% confidence interval (CI) were calculated and then converted back to the correlation coefficient (r) scale for reporting. Heterogeneity between studies was assessed using Cochran's Q test and I^2^ statistics [9]. On the basis of this heterogeneity assessment, DerSimonian‐Laird random‐effects models were applied when I^2^ exceeded 50% or when prespecified clinical or methodological heterogeneity, such as differences in specimen types (e.g., peripheral blood, PBMC, and skin samples), assay methods, or outcome definitions, rendered a common‐effect assumption inappropriate. Conversely, fixed‐effect models were used when neither condition applied [8]. All analyses were conducted in R version 4.4.1 using the metafor package, with manual calculations in Microsoft Excel, while between‐study variance in random‐effects models was estimated using the DerSimonian‐Laird method. All statistical analyses were two‐tailed, with a significance level set at *p* < 0.05. Because most biomarkers contributed only 2–4 effect sizes, subgroup analyses, meta‐regression, funnel plots, and Egger's regression tests were not conducted. The four‐stage pathological framework was employed solely for descriptive and conceptual evidence integration, and no stage‐specific pooled effect estimates were computed.

## 3 Results

Literature Screening Process and Characteristics of Included Studies.

A total of 106 articles were initially retrieved. After two rounds of screening, six studies met the inclusion criteria and were included in the meta‐analysis for different biomarkers (Figure 1) [7, 10, 11, 12, 13, 14]. The detailed characteristics of the included studies are presented in Table 1.

**Figure 1:**
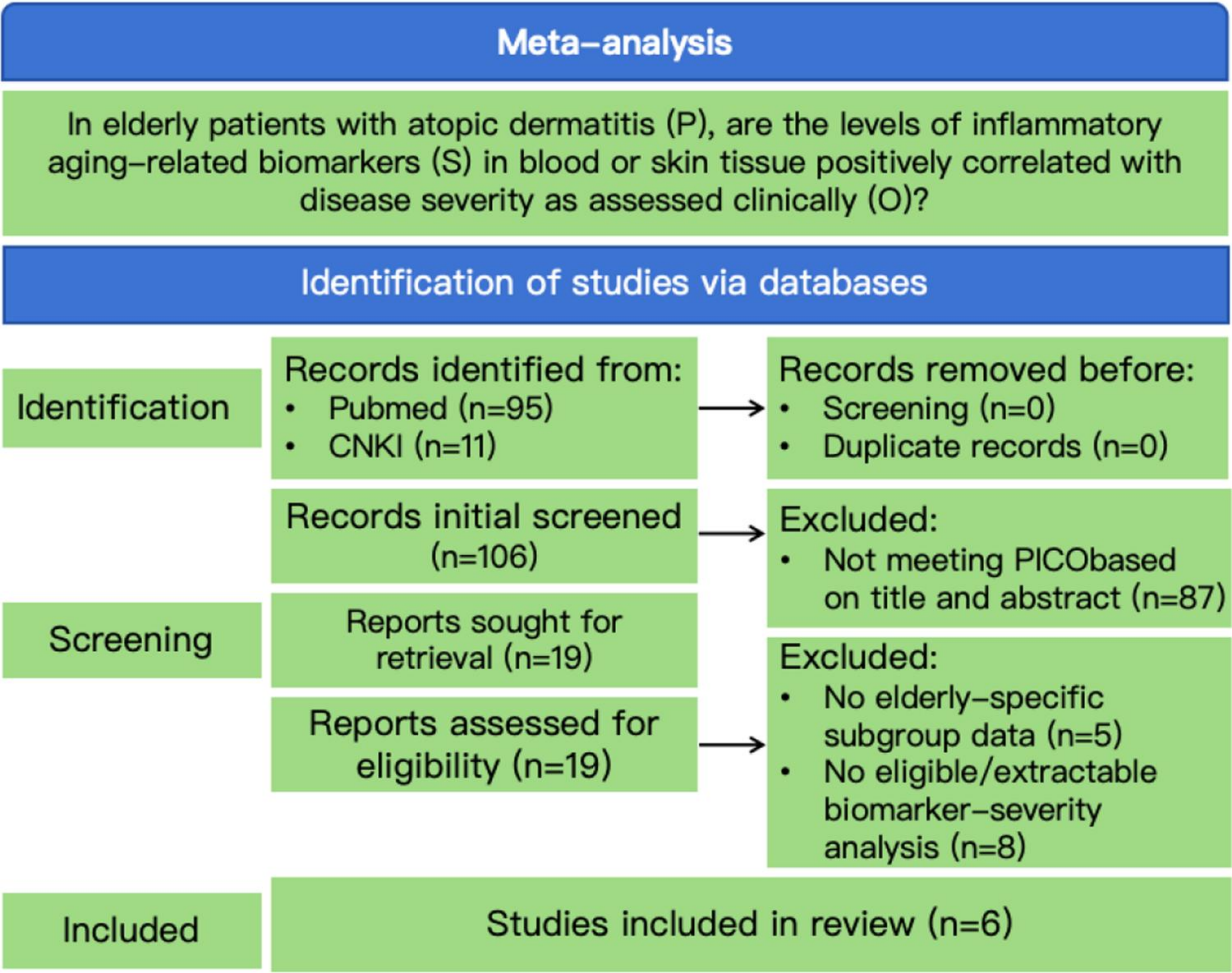
PRISMA flow diagram illustrating the study selection process. Schematic diagram indicating the study selection process based on the PRISMA guidelines. It shows the total number of records identified from PubMed and CNKI databases, the number of duplicates removed, the records excluded during screening, and the final number of studies included in the review. A total of six studies met the eligibility criteria for the meta‐analysis.

**Table 1:** Characteristics of studies included in the meta‐analysis.

Published	Original r value	Original *p* value	Sample type	Biomarker	AD severity/activity measure	Sample (*n*)
Zhou 2019	0.521	0.041	Skin	CCL26	SCORAD	27
Zhao 2024	0.4736	0.0006	Peripheral blood	Total IgE	SCORAD	98
Zhao 2024	0.5011	0.0001	Peripheral blood	EOS	SCORAD	98
Zhao 2024	0.7071	0.0001	Peripheral blood	IL‐4	SCORAD	98
Zhao 2024	0.7643	0.0001	Peripheral blood	IL‐17	SCORAD	98
Jia 2023	0.607	0.0010	Peripheral blood	EOS	SCCA expression level	25
Jia 2023	0.105	0.616	Peripheral blood	Total IgE	SCCA expression level	25
Fang 2024	0.733	0.025	PBMC/Peripheral blood	Total IgE	SCORAD	9
Fang 2024	0.786	0.036	Skin	Total IgE	SCORAD	7
Wang 2020	0.38	0.0010	Peripheral blood	IL‐4	SCORAD	56
Wang 2020	0.37	0.0010	Peripheral blood	TARC	SCORAD	56
Wang 2020	0.47	0.0010	Peripheral blood	TSLP	SCORAD	56
Wang 2020	0.33	0.0010	Peripheral blood	IL‐17A	SCORAD	56
Wang 2020	0.32	0.0010	Peripheral blood	IL‐22	SCORAD	56
He 2020	0.57	0.0068	Peripheral blood	MMP12	SCORAD	21
He 2020	0.59	0.0053	Peripheral blood	EZR	SCORAD	21
He 2020	0.56	0.0082	Peripheral blood	CDH3	SCORAD	21
He 2020	0.45	0.0042	Peripheral blood	CCL7	SCORAD	21
He 2020	0.49	0.025	Peripheral blood	SELE	SCORAD	21
He 2020	0.39	0.079	Peripheral blood	CCL13	SCORAD	21
He 2020	0.49	0.024	Peripheral blood	CCL20	SCORAD	21
He 2020	0.51	0.019	Peripheral blood	CXCL1	SCORAD	21
He 2020	0.54	0.011	Peripheral blood	IL‐6	SCORAD	21
He 2020	0.49	0.025	Peripheral blood	IL‐17C	SCORAD	21

*Note:* This table presents the characteristics of the six studies included in the meta‐analysis, including the first author, publication year, sample size, biomarker(s) analyzed, source of the sample (peripheral blood or skin), and the disease severity assessment tool (SCORAD or SCCA expression).

### 3.1 Meta‐Analysis of Inflammatory Biomarkers and Disease Severity

Total IgE: Three publications contributed four effect estimates (total *N* = 139) to the analysis (Table 2), which Fang 2024 contributed two nonoverlapping participant datasets (PBMC/peripheral blood, *n* = 9; skin, *n* = 7) and was counted as one publication. Random‐effects model meta‐analysis revealed a moderate positive correlation between total IgE levels and disease severity (pooled r = 0.464, 95% CI: 0.162–0.687) (Figure 2A). The heterogeneity test showed moderate heterogeneity (I^2^ = 48.2%, *p* = 0.122), indicating some variability in the strength of association across different studies (Tables 3 and 4). This suggests that IgE still reflects disease activity in elderly AD, but with some heterogeneity. Given the potential concern that SCCA‐based assessment might not be directly comparable with standard clinical severity scores, we tested the robustness of this finding by repeating the meta‐analysis after excluding the study that used this surrogate measure (Jia et al., 2023). The recalculated pooled correlation was r = 0.514 (95% CI, 0.344–0.651, I^2^ = 2.1%), which remained consistent with the primary analysis, suggesting that the observed association was not driven by this surrogate measure.

**Table 2:** Summary of original Pearson r and Zr Transformation for inflammatory biomarkers and disease severity.

Biomarker subgroup (effect‐estimate count, k)	Study	r	*n*	Severity/activity measure	Sample type	Zr	Vz
Total IgE subgroup (k = 4)	Zhao 2024	0.4736	98	SCORAD	Peripheral blood	0.5147	0.01053
	Jia 2023	0.105	25	SCCA	Peripheral blood	0.1054	0.04545
	Fang 2024 (PBMC)	0.733	9	SCORAD	Peripheral blood	0.9352	0.1667
	Fang 2024 (Skin)	0.786	7	SCORAD	Skin	1.0609	0.25
EOS subgroup (k = 2)	Zhao 2024	0.5011	98	SCORAD	Peripheral blood	0.5508	0.01053
	Jia 2023	0.607	25	SCCA	Peripheral blood	0.7042	0.04545
IL‐4 subgroup (k = 2)	Zhao 2024	0.7071	98	SCORAD	Peripheral blood	0.8814	0.01053
	Wang 2020	0.38	56	SCORAD	Peripheral blood	0.4001	0.01887
IL‐17 subgroup (k = 3)	Zhao 2024	0.7643	98	SCORAD	Peripheral blood	1.0065	0.01053
	Wang 2020	0.33	56	SCORAD	Peripheral blood	0.3428	0.01887
	He 2020	0.49	21	SCORAD	Peripheral blood	0.5361	0.05556

*Note:* This table summarizes the pooled correlation coefficients (r) for each inflammatory biomarker and their corresponding Zr values (Fisher's Z transformation). The table presents the 95% CI for both r and Zr, as well as the heterogeneity statistics (I^2^) for each biomarker group. The r is the original correlation coefficient. Zr is used for statistical aggregation and to facilitate more accurate comparisons across studies. The conversion from r to Zr is performed using the Fisher's Z transformation formula: Zr = 0.5 * ln((1 + r)/(1 ‐ r)), Vz = 1/(*n* − 3).

**Figure 2:**
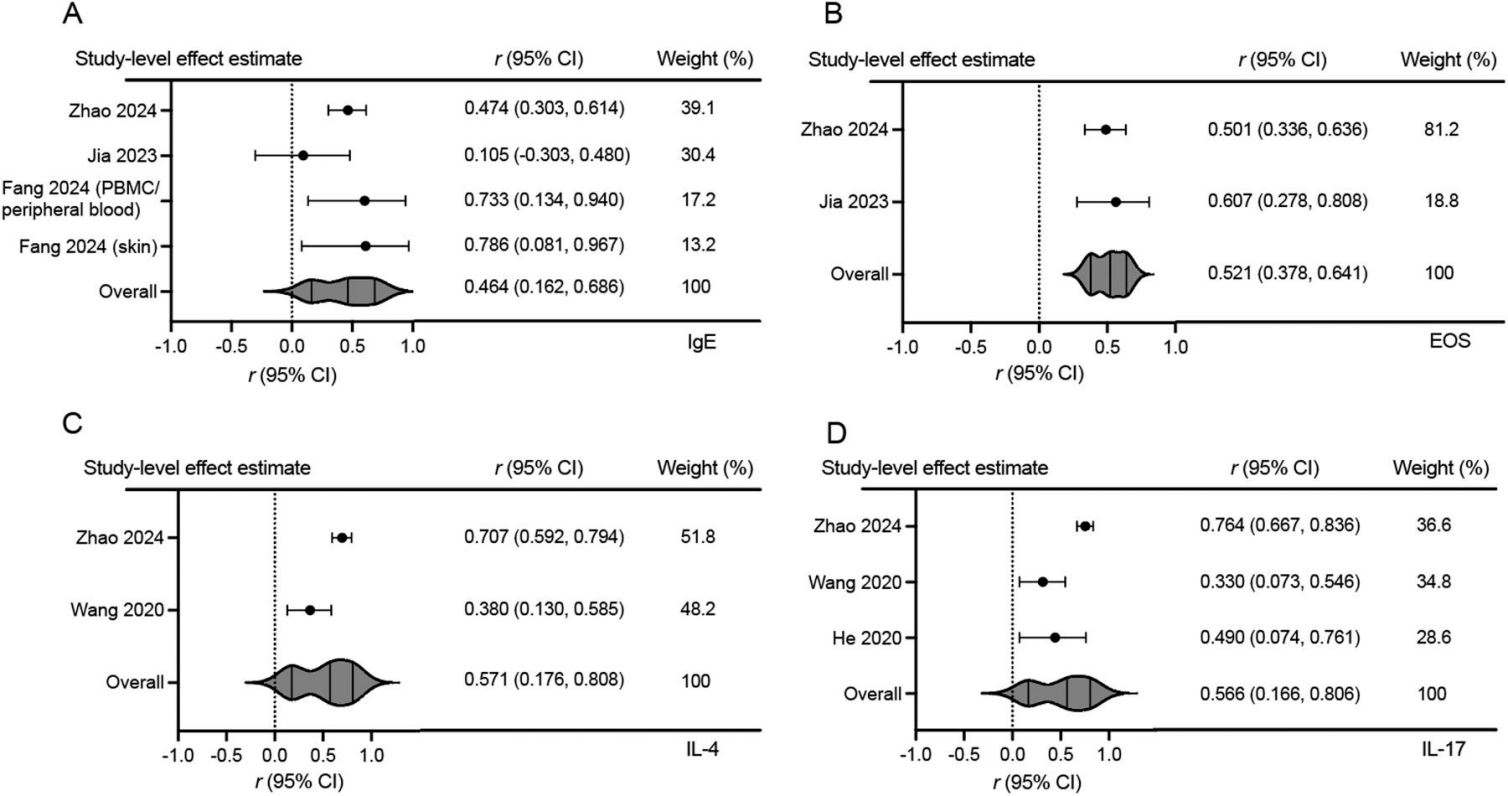
Forest plots of correlations between inflammatory biomarkers and AD disease severity or activity in older adults. The forest plots illustrate the associations of (A) total IgE, (B) eosinophils (EOS), (C) IL‐4, and (D) IL‐17 with AD disease severity. In each plot, individual points denote study‐level effect estimates, with their sizes reflecting the weight assigned in the meta‐analysis and the horizontal lines representing the corresponding 95% CIs. Since the original studies reported correlation coefficients (r) without providing 95% CIs, we calculated the study‐level CIs from the reported r values and sample sizes using Fisher's z transformation. Violin plots indicate the pooled estimates and their associated 95% CIs. All analyses were performed on the Fisher's z scale, and both study‐level and pooled estimates were subsequently back‐transformed to r for presentation. DerSimonian‐Laird random‐effects models were applied for total IgE, IL‐4, and IL‐17, whereas a fixed‐effect model was used for EOS based on the heterogeneity assessment. The horizontal axis is labelled as r and is constrained to its theoretical range of −1 to +1. Besides, SCCA expression was treated as a laboratory surrogate of disease activity rather than as a validated clinical severity score.

**Table 3:** Heterogeneity assessment for pooled correlations of inflammatory biomarkers and disease severity.

Subgroup	Cochran's Q (*p*‐value)	Higgins I^2^	Heterogeneity assessment	Basis for model selection
Total IgE	5.794 (*p* = 0.122)	0.4823	Moderate heterogeneity, statistically nonsignificant	Random‐effects model adopted because of prespecified clinical/methodological heterogeneity
EOS	0.420 (*p* = 0.517)	0	No heterogeneity	Fixed‐effects model adopted
IL‐4	7.881 (*p* = 0.005)	0.8731	High heterogeneity	Random‐effects model adopted
IL‐17	15.853 (*p* < 0.001)	0.8738	High heterogeneity	Random‐effects model adopted

*Note:* This table provides an assessment of heterogeneity for each biomarker's pooled correlation. For each biomarker, Cochran's Q statistic, its corresponding *p*‐value, and the I^2^ index are reported to quantify between‐study heterogeneity. The selection of either a random‐effects or fixed‐effect model was guided by both the I^2^ value and prespecified clinical or methodological heterogeneity (e.g., differences in specimen types or outcome measures). Accordingly, a random‐effects model was applied for total IgE despite its I^2^ below 50%, whereas a fixed‐effect model was used for eosinophils given the negligible heterogeneity. Cochran's Q and I^2^ are used as: Q = Σ[W_i × (Y_i ‐ Ŷ)^2^], I^2^ = [(Q ‐ df)/Q] × 100%.

**Table 4:** Summary of pooled correlation coefficients for inflammatory biomarkers and disease severity.

Biomarker	No. of effect estimates (k)	Total sample size (*N*)	Assessment tools (effect‐estimate count)	Pooled r (95% CI)	I^2^ (%)	*p* (heterogeneity)	Effect model	Conclusion
Total IgE	4	139	SCORAD (3), SCCA (1)	0.464 (0.162 to 0.687)	48.2	0.122	Random‐effects	Weak‐to‐moderate positive correlation; moderate heterogeneity and a wide confidence interval.
EOS	2	123	SCORAD (1), SCCA (1)	0.522 (0.379 to 0.642)	0	0.517	Fixed‐effects	Moderate positive correlation; low heterogeneity; relatively robust result.
IL‐4	2	154	SCORAD (2)	0.571 (0.176 to 0.808)	87.3	0.0050	Random‐effects	Moderate positive correlation; extremely high heterogeneity; unstable finding.
IL‐17	3	175	SCORAD (3)	0.566 (0.165 to 0.806)	87.4	< 0.001	Random‐effects	Moderate‐to‐strong positive correlation; high heterogeneity may reflect both biological and methodological differences.

*Note:* This table summarizes the primary pooled correlation coefficients, 95% CIs, heterogeneity statistics, effect models, and interpretations. Formula for converting Fisher's Z back to r: r = [exp(2×Zr)−1]/[exp(2×Zr)+1].

Eosinophils (EOS): Two studies (*n* = 123) were included in the analysis (Table 2). Fixed‐effect model analysis showed a stable moderate positive correlation between eosinophil counts and disease severity (pooled r = 0.522, 95% CI: 0.379–0.642), with almost no heterogeneity (I^2^ = 0%, *p* = 0.517) (Figure 2B, Tables 3 and 4). This suggests that eosinophils remain a stable indicator of inflammation in older adults with AD. It should be noted, however, that the SCCA‐based study (Jia et al., 2023) was also included in this analysis, and no sensitivity analysis could be performed given the limited number of available studies.

IL‐4: Two studies (*n* = 154) were included in the analysis (Table 2). Due to very high heterogeneity (I^2^ = 87.3%, *p* = 0.005), a random‐effects model was used (Tables 3 and 4). The results showed a moderate positive correlation between IL‐4 levels and disease severity of AD (pooled r = 0.571, 95% CI: 0.176–0.808), but with a wide confidence interval, indicating instability (Figure 2C).

IL‐17: Three studies (*n* = 175) were included in the analysis (Table 2). There was also high heterogeneity (I^2^ = 87.4%, *p* < 0.001), so a random‐effects model was used (Table 3 and Table 4). The results showed a moderate to strong positive correlation between IL‐17 (including IL‐17A and IL‐17C) and disease severity of AD (pooled r = 0.566, 95% CI: 0.165–0.806) (Figure 2D). The strength of the association and the heterogeneity suggest variability in IL‐17‐axis involvement among older adults with AD.

Both IL‐4 and IL‐17 exhibited moderate to strong correlations with disease severity, suggesting their potential involvement in the immunopathological mechanisms of the disease. However, substantial heterogeneity was observed, which may reflect biological or methodological differences and does not by itself establish distinct immune endophenotypes.

### 3.2 Pathological Stage‐Based Integration Analysis

For descriptive evidence integration, biomarkers were organized into four proposed conceptual stages according to their putative roles in the immune response (Table 5). This organization summarizes study‐level associations and does not represent a stage‐specific meta‐analysis or an established temporal sequence. According to that, we aimed to assess the overall correlation strength between each stage of the dynamic process, from the systemic inflammation to local tissue dysfunction, and disease severity.

**Table 5:** Study‐level correlation coefficients of inflammatory biomarkers with disease severity in elderly atopic dermatitis, organized by proposed pathophysiological stages.

Study	Step	Biomarker	r	*n*	*p* value
He 2020	Step 1: Inflammaging	IL‐6	0.54	21	0.011
He 2020	Step 1: Inflammaging	CXCL1	0.51	21	0.019
He 2020	Step 1: Inflammaging	CCL7	0.45	21	0.0042
He 2020	Step 1: Inflammaging	SELE	0.49	21	0.025
Zhao 2024	Step 2: Abnormally amplified Th2 response under inflammatory conditions	IL‐4	0.7071	98	0.0001
Wang 2020	Step 2: Abnormally amplified Th2 response under inflammatory conditions	IL‐4	0.38	56	0.0010
Wang 2020	Step 2: Abnormally amplified Th2 response under inflammatory conditions	TSLP	0.47	56	0.0010
Wang 2020	Step 2: Abnormally amplified Th2 response under inflammatory conditions	TARC	0.37	56	0.0010
Zhou 2019	Step 2: Abnormally amplified Th2 response under inflammatory conditions	CCL26	0.521	27	0.041
He 2020	Step 2: Abnormally amplified Th2 response under inflammatory conditions	CCL13	0.39	21	0.079
Zhao 2024	Step 2: Abnormally amplified Th2 response under inflammatory conditions	Total IgE	0.4736	98	0.0006
Zhao 2024	Step 2: Abnormally amplified Th2 response under inflammatory conditions	EOS	0.5011	98	0.0001
Jia 2023	Step 2: Abnormally amplified Th2 response under inflammatory conditions	EOS	0.607	25	0.0010
Jia 2023	Step 2: Abnormally amplified Th2 response under inflammatory conditions	Total IgE	0.105	25	0.616
Fang 2024	Step 2: Abnormally amplified Th2 response under inflammatory conditions	Total IgE	0.733	9	0.025
Fang 2024	Step 2: Abnormally amplified Th2 response under inflammatory conditions	Total IgE	0.786	7	0.036
Zhao 2024	Step 3: Aberrant involvement of the Th17/IL‐17 axis	IL‐17	0.7643	98	0.0001
Wang 2020	Step 3: Aberrant involvement of the Th17/IL‐17 axis	IL‐17A	0.33	56	0.0010
He 2020	Step 3: Aberrant involvement of the Th17/IL‐17 axis	IL‐17C	0.49	21	0.025
Wang 2020	Step 3: Aberrant involvement of the Th17/IL‐17 axis	IL‐22	0.32	56	0.0010
He 2020	Step 3: Aberrant involvement of the Th17/IL‐17 axis	CCL20	0.49	21	0.024
He 2020	Step 4: Barrier fails to restore due to impaired tissue remodeling	MMP12	0.57	21	0.0068
He 2020	Step 4: Barrier fails to restore due to impaired tissue remodeling	EZR	0.59	21	0.0053
He 2020	Step 4: Barrier fails to restore due to impaired tissue remodeling	CDH3	0.56	21	0.0082

*Note:* This table presents the correlation coefficients (r) between various inflammatory biomarkers and disease severity in elderly patients with atopic dermatitis, as originally reported in each included study. Biomarkers are grouped into four conceptual stages according to their proposed roles in disease pathogenesis. All data are study‐level descriptive summaries; no formal meta‐analysis was performed across biomarkers within any stage.

Inflammaging Stage: This stage represents the upstream immune micro‐environment for disease onset. The integrated analysis shows that non‐specific inflammation and endothelial activation markers, such as IL‐6 [15, 16], CXCL1 [17, 18], CCL7 [19], and SELE, are strongly positively correlated with disease severity in elderly AD (r range: 0.45–0.54) (Tables 5 and 6). These findings provide quantitative evidence of a significant “inflammaging” state in this population, which suggest those chronic, low‐grade systemic inflammation not only is associated with disease pathogenesis but may correlate with tissue sensitivity, potentially creating a pro‐inflammatory milieu that could facilitate the subsequent aberrant amplification of specific immune responses.

**Table 6:** Descriptive summary of study‐level correlation ranges by proposed pathophysiological stage.

Pathological step	Key biomarkers involved	No. of studies	Total sample size	Strength of association (r range)	Statistical evidence	Interpretation
Step 1: Inflammaging	IL‐6, CXCL1, CCL7, SELE	1	21	0.45–0.54	All correlations significant (*p* < 0.05)	Multiple inflammaging‐associated markers show moderate‐to‐strong correlations with disease severity within the same cohort, consistent with an early pro‐inflammatory state.
Step 2: Abnormally amplified Th2 response under inflammatory conditions	IL‐4, TSLP, TARC, CCL26, CCL13, EOS, Total IgE	6	243	0.10–0.79	10 of 12 correlations significant; biomarker‐specific meta‐analyses reported separately	The observed associations are consistent with Th2‐skewed immune activity; variability may reflect biological and methodological differences across cohorts.
Step 3: Aberrant involvement of the Th17/IL‐17 axis	IL‐17, IL‐17A, IL‐17C, IL‐22, CCL20	3	175	0.32–0.76	Predominantly significant; family‐level consistency	Th17‐related mediators are associated with disease severity, consistent with proposed involvement of the IL‐17 axis; causal direction cannot be inferred.
Step 4: Barrier fails to restore due to impaired tissue remodeling	MMP12, EZR, CDH3	1	21	0.56–0.59	All correlations significant (*p* < 0.01)	Correlations among remodeling‐related molecules are consistent with impaired barrier restoration, but causal direction cannot be inferred.

*Note:* This table provides a stage‐level descriptive summary of the study‐level correlation coefficients presented in Table 5. Study counts refer to individual publications; stage‐level sample sizes are summed across nonoverlapping eligible datasets; and r values represent descriptive ranges of study‐level effects, not stage‐specific pooled meta‐analytic estimates. Correlation strength was interpreted as follows: strong (r ≥ 0.5), moderate (0.3 ≤ r < 0.5), and weak (r < 0.3). The pathological stages are conceptualized based on current understanding of AD immunopathogenesis and are used for descriptive evidence integration purposes.

Th2 Immune Amplification Stage: In this phase, we integrated core Th2 cytokines, including IL‐4, associated chemokines (TARC, CCL26, CCL13), and downstream effectors such as total IgE and eosinophil levels [20]. Analysis revealed moderate to strong positive correlations between this biomarker cluster and disease severity (r range: 0.10–0.79), consistent with continued Th2‐related immune activity in older adults with AD (Tables 5 and 6). However, it is noteworthy that the heterogeneity was observed across studies that the strength of the correlation between IL‐4 and IgE varied considerably (Figure 2). These findings suggest that although Th2‐mediated immune responses persist in the low‐grade inflammaging, their expression and magnitude may have shifted, potentially reflecting evolving immune dynamics or the presence of distinct disease subtypes. Meanwhile, because of effect sizes for IL‐4 and total IgE, methodological differences also need to be considered.

Th17 Axis Abnormal Intervention Stage: At this stage, we assessed Th17‐associated biomarkers, encompassing IL‐17, IL‐17A, IL‐17C, CCL20, and IL‐22 [21]. These markers showed moderate or strong positive correlations with disease severity (r range: 0.32‐0.76), consistent with possible involvement of the Th17 pathway in older adults with AD (Tables 5 and 6). Besides, the high heterogeneity (I^2^ = 87.4% of IL‐17, including IL‐17, IL‐17A, IL‐17C) is likely of substantial pathophysiological relevance, may reflecting a hypothesis that a subset of patients exhibits a prominent “Th17‐high” profile, whereas others remain primarily Th2‐driven (Table 4). However, besides diverse clinical presentations, methodological differences may also trigger the high heterogeneity.

Tissue Damage and Repair Imbalance Stage: This proposed domain focuses on tissue remodeling and disease chronicity, involving barrier‐related molecules such as MMP12, EZR, and CDH3 (data from He et al., 2020) (Table 5) [10, 22]. The integrated analysis showed strong correlations between these biomarkers and disease severity (r range: 0.56‐0.59) (Tables 5 and 6). These correlations are consistent with an association between tissue‐remodeling abnormalities and disease severity. The upregulation of protease activity, particularly MMP12, is a key molecular event connecting immune inflammation with keratinocyte differentiation disorder and extracellular matrix degradation, marking the onset of the inflammation‐barrier destruction vicious cycle and the chronicization of the disease.

Across the four proposed stages, we observed a consistent pattern of positive associations between biomarker levels and disease severity. This descriptive organization provides a hypothesis‐generating framework linking inflammaging (stage 1), Th2 responses (stage 2), Th17‐axis involvement (stage 3), and impaired barrier repair (stage 4) in older adults with AD pathogenesis. However, given the predominantly cross‐sectional design of the included studies, this framework is hypothesis‐generating in nature, and the available evidence does not permit conclusions regarding directionality, temporal progression, or a causal biological trajectory.

### 3.3 Investigations of Heterogeneity and Reporting Bias

Formal subgroup analyses and meta‐regression to investigate sources of heterogeneity were not performed because the number of studies contributing to each meta‐analysis was too small (2–4 effect estimates) to yield meaningful or reliable results. Similarly, publication bias was not formally assessed using funnel plots or Egger's regression tests, as the small number of studies per outcome (*n* < 10) would render such analyses statistically underpowered. These limitations are acknowledged in the Discussion.

In addition, quality assessment results are summarized in Supplementary file. All studies had clear objectives. Most used validated clinical severity measures, whereas Jia et al. used SCCA as a laboratory surrogate of disease activity. Common limitations included insufficient confounding control, small sample sizes, and incomplete reporting of sampling or response rates. No study was excluded on quality grounds, though these limitations informed result interpretation.

## 4 Discussion

This meta‐analysis further quantitatively assessed the correlation between key immune biomarkers and disease severity in elderly AD. Based on these findings, we propose a hypothesis‐generating framework that organizes the observed associations across inflammaging‐related, Th2, Th17, and tissue‐remodeling domains. The results illustrate the complexity of immune dysregulation in older adults with AD.

These findings support and refine a conceptual framework for the chronicity of elderly AD. Notably, Stage 1 biomarkers including IL‐6 and CXCL1 exhibited a strong correlation with disease severity, echoing recent observations that serum IL‐6 levels serve as sensitive indicators of immunocompetence in older adults [23]. This age‐associated, chronic low‐grade systemic inflammation may help to establish a hyperresponsive immune microenvironment that likely recalibrates the threshold for skin immune homeostasis. By lowering this threshold, inflammaging increases susceptibility to both endogenous and exogenous triggers [24]. Thereby, inflammaging may serve as a critical background and catalyst for the initiation and chronicity of AD in older adults [25, 26].

Recent studies on late‐onset atopic dermatitis (LOAD) provide an important context for the proposed Stage 2. Unlike pediatric forms dominated by Th2 responses, LOAD frequently presents as intrinsic AD with lower IgE levels and distinct inflammatory profiles [23, 27, 28]. Our analysis of the Th2 immune axis revealed its evolving characteristics in elderly AD. Although the Th2 pathway overall maintained a moderate positive correlation with severity, there were significant differences in the performance among its components. Eosinophils maintained the most stable and consistent association, supporting their potential reliability as indicators. However, the weaker correlation of total IgE appears consistent with observations that intrinsic AD variants, which are more prevalent in elderly populations, may include patients with Th1‐dominant rather than Th2‐skewed responses. Moreover, IL‐4 exhibited substantial heterogeneity (I^2^ = 87.3%), which may reflect distinct Th2 response phenotypes. This potential phenotypic diversity is consistent with the findings of Suárez‐Fariñas et al., who demonstrated that intrinsic AD shows similar Th2 but higher Th17 activation compared with extrinsic variants [29]. Some patients may retain a “Th2‐high” profile typical of younger cohorts, whereas others may exhibit “Th2‐evolved” pattern. In the “Th2‐evolved” group, IL‐4 signals may attenuate and disease becomes more dependent on alternative cytokines such as IL‐13 or IL‐31. Consistent with Gittler et al. observed that acute AD onset involves parallel activation of Th2 and Th22 cytokines without significant Th1 switching, challenging the traditional biphasic model [30]. This potential phenotypic diversity provides an explanation for the variable clinical responses to traditional Th2‐targeted therapies in the older adults with AD.

Meanwhile, immune aging‐related functional decline in immune regulation networks may weaken the mutual constraints between immune pathways, potentially allowing the normally secondary Th17 axis to be abnormally activated and intervene (Stage 3) [31]. However, analysis of the Th17/IL‐17 axis revealed substantial heterogeneity (I^2^ = 87.4%), suggesting considerable variability across studies. Because only two to four effect estimates were available for most biomarkers, subgroup analysis, meta‐regression, funnel plots, and Egger's tests were not performed, as these analyses would be unreliable or underpowered. This heterogeneity is biologically interesting and may point to underlying pathophysiological diversity, but it may also be influenced by methodological differences such as variations in patient characteristics, AD phenotypes, severity instruments, assay platforms, and sample types. Although pooled data demonstrated a robust overall correlation, individual studies reported widely divergent effect sizes (e.g., Zhao 2024: r = 0.764; Wang 2020: r = 0.33). These discrepancies raise the hypothesis that older adults with AD may encompass distinct immune profiles, with some patients exhibiting a “Th17‐high” pattern and others showing minimal Th17 pathway activation. It is plausible that a subset of older adults with AD may shift from a “Th2‐dominant” to a “Th2/Th17‐mixed” or even “Th17‐dominant” immune pattern, potentially influenced by individual variations in immune dysregulation, skin barrier integrity, and microbiome composition. Furthermore, this heterogeneity is consistent with observed clinical diversity and may help explain differential responses to biologic therapies targeting IL‐17 or IL‐23. However, we acknowledge that these interpretations remain hypothetical and require validation in prospective or molecularly stratified cohorts.

Stage 4 involves sustained mixed inflammation severely interfering with keratinocyte function and innate reparative capacity. Previous investigations highlight matrix metalloproteinases, especially MMP‐9 and MMP‐12, as critical mediators of tissue remodeling in chronic inflammatory skin conditions [32, 33]. Elevated MMP activity disrupts normal skin architecture and exacerbates barrier dysfunction, establishing a self‐perpetuating “inflammation‐barrier destruction” cycle [30, 34]. In our analysis, biomarkers associated with tissue remodeling (such as MMP12) demonstrated the strongest correlation with disease severity, consistent with observations that MMP12 expression distinguishes allergic conditions across the atopic march spectrum. The activation of MMP12 signifies transition into aberrant tissue remodeling states, regardless of upstream immune polarization. The convergence on defective structural repair may provide biological explanation for refractory, non‐healing lesions commonly observed in older adults wth AD. Importantly, Agache et al. emphasize that therapeutic decisions in older adults must consider biological age, frailty, and immunosenescence status rather than disease severity alone [23]. The limited availability of age‐specific evidence for biologic therapies complicates treatment selection, despite there are some special cases that promising results with dupilumab (IL‐4/IL‐13 blockade) in patients over 65 years [35].

In conclusion, our findings support a dynamic, multifactorial conceptual model of AD in older adults in which inflammaging‐related, Th2, Th17, and tissue‐remodeling signals may overlap. These associations are consistent with, but do not prove, a contribution to impaired barrier repair and disease chronicity. This hypothesis‐generating framework supports further evaluation of biomarker‐defined subgroups and barrier‐restoration strategies in prospective studies.

Otherwise, several limitations warrant acknowledgment. We quantitatively synthesized only four biomarkers, total IgE, eosinophils, IL‐4, and IL‐17, and did not examine other potentially relevant inflammatory mediators. The cross‐sectional design of most included studies means we cannot infer causality or determine which comes first in the observed associations. With only two to four effect sizes per biomarker, we could not formally test for publication bias using funnel plots or Egger's tests, nor could we run subgroup analyses or meta‐regression to explore heterogeneity in any meaningful way. Moreover, the literature search was limited to PubMed and CNKI, so relevant studies indexed exclusively in other databases may have been missed. We also tried to consider both biological and methodological sources of heterogeneity, but the small number of studies made it difficult to systematically evaluate how methodological differences, such as patient characteristics, AD phenotyping, severity scales, laboratory methods, or sample type, might have influenced the results. Lastly, because the evidence base is still limited, our four‐stage model should be taken as a hypothesis‐generating proposal rather than a proven pathway. Clearly, larger prospective cohort studies with standardized protocols are needed to validate this model and to test whether biomarker‐based subgroups could eventually guide clinical management.

## Author Contributions

**Nan Wu:** writing – original draft. **Yingjie Li:** data curation. **Xiaojie Wang:** data curation. **Yangyang Wu:** writing – review and editing. **Wenhao Yin:** writing – review and editing.

## Conflicts of Interest

The authors declare no conflicts of interest.

## Supporting information


Supporting File

## Data Availability

The data that support the findings of this study are available from the corresponding author upon reasonable request. All data and analysis code supporting the findings of this study are provided in the Supplementary File.
