# Supplementary material for: Inflammatory Aging‐Driven Immune Axis Imbalance and the Mechanisms of Chronicity in Elderly Atopic Dermatitis: A Systematic Review and Meta‐Analysis
Source: Immun Inflamm Dis. 2026 Sep 29;14(9):e70528. doi: 10.1002/iid3.70528 (PMC13623012; doi:10.1002/iid3.70528)
Supplement: Supplementary file 1 [file IID3-14-e70528-s001.docx]

**Supplemental file**

**Inflammatory Aging-Driven Immune Axis Imbalance and the Mechanisms of Chronicity in Elderly Atopic Dermatitis: A Systematic Review and Meta-Analysis**

**1 Reconciliation of Study Counts and Screening Flow**

**1.1 Search Strategy**

The protocol of our work was registered with the International Prospective Register of Systematic Reviews (PROSPERO) under the registration number CRD420261325988 (available at https://www.crd.york.ac.uk/prospero/). The reporting of this study follows the Preferred Reporting Items for Systematic Reviews and Meta-Analyses (PRISMA) guidelines. In detail, the scientific question was formulated using a well-defined P–S–O framework: the population (P) consisted of elderly patients with AD, the study factor (S) encompassed inflammaging-related biomarkers, and the outcome (O) was the clinical quantitative assessment of disease severity. And the objective of this study was to systematically synthesize extant literature to evaluate the correlation between inflammaging-related biomarker levels and disease severity in the elderly AD population.

A comprehensive systematic search was performed across PubMed and the China National Knowledge Infrastructure (CNKI) from inception through December 15, 2025. No language restrictions were applied, and reference lists of eligible reports were manually screened. In PubMed, a strategy combining Medical Subject Headings (MeSH) terms and free-text keywords was utilized. Search terms included: (1) Disease-related: "Dermatitis, Atopic," "Atopic Dermatitis," and "Eczema." (2) Population-related: "Aged," "Aging," "Geriatric," "Elderly," and "older adult." (3) Biomarker-related: "Inflammation," "Biomarkers," "Cytokines," and "Chemokines." (4) Outcome-related: "Severity of Illness Index," "Disease Progression," "Eczema Area and Severity Index (EASI)," and " Scoring Atopic Dermatitis (SCORAD) ". These terms were integrated using Boolean operators (and/or) to maximize retrieval sensitivity.

For the Chinese CNKI database, index terms such as "atopic dermatitis" and "elderly" were used, supplemented by full-text searches for "EASI," "SCORAD," "scoring", and "severity" in Chinese, thereby capturing data from the Chinese population on the clinical characteristics and inflammatory profiles of elderly AD populations. Furthermore, reference lists of all identified articles were manually screened to minimize potential omissions.

**1.2 Reconciled Screening Counts**

| **Screening stage** | **PubMed** | **CNKI** | **Total** |
| --- | --- | --- | --- |
| Records identified | 95 | 11 | 106 |
| Excluded during initial screening | 83 | 4 | 87 |
| Reports assessed at full text | 12 | 7 | 19 |
| Reports excluded after full-text review | 9 | 4 | 13 |
| Studies included in the review | 3 | 3 | 6 |

Summary: A total of 106 records were identified (95 from PubMed and 11 from CNKI). After initial screening of title and abstract check, 19 reports underwent full-text assessment, which 13 were excluded and 6 studies were included. Therefore, the PRISMA sequence is 106 to 19 to 6, 6 at final stage for meta analysis.

**1.3 Six Studies Included after Full-Text Screening**

| **ID** | **Source** | **Original no.** | **Citation** | **Status/notes** |
| --- | --- | --- | --- | --- |
| 1 | PubMed | 20 | He H, et al. Increased cardiovascular and atherosclerosis markers in blood of older patients with atopic dermatitis. Ann Allergy Asthma Immunol. 2020;124(1):70-78. PMID: 31622668. | Passed initial and full-text screening |
| 2 | PubMed | 36 | Zhou L, et al. Age-specific changes in the molecular phenotype of patients with moderate-to-severe atopic dermatitis. J Allergy Clin Immunol. 2019;144(1):144-156. PMID: 30685456. | Passed initial and full-text screening |
| 3 | PubMed | 95 | Wang S, Zhu R, Gu C, et al. Distinct clinical features and serum cytokine pattern of elderly atopic dermatitis in China. J Eur Acad Dermatol Venereol. 2020;34(10):2346-2352. PMID: 32163633. | Passed initial and full-text screening |
| 4 | CNKI | 5 | Zhao P, Yu C, Dou Y, et al. Clinical analysis of 108 older patients with atopic dermatitis. Journal of Clinical Dermatology. 2024;53(6):330-333. | Passed initial and full-text screening |
| 5 | CNKI | 6 | Fang X. Role and mechanism of natural killer cells in elderly atopic dermatitis [dissertation]. Peking Union Medical College, 2024. DOI:10.27648/d.cnki.gzxhu.2024.000009. | Passed initial and full-text screening |
| 6 | CNKI | 9 | Jia H, Wan H, Zhang D. Changes and significance of peripheral blood Th1, Th2, and Th17 cells and squamous cell carcinoma antigen levels in older patients with atopic dermatitis. China Journal of Modern Medicine. 2023;33(19):85-91. | Passed initial and full-text screening |

Composition of the 19 full-text reports: 13 reports that passed initial screening but were excluded after full-text review (PubMed records 1, 2, 3, 4, 11, 12, 13, 25, and 34; CNKI records 4, 7, 10, and 11), plus the 6 studies that passed full-text screening to do the meta analysis.

**1.4 Thirteen Reports Excluded after Full-Text Review**

| **ID** | **Source** | **Original no.** | **Citation** | **Single primary exclusion reason** | **Basis in screening notes** |
| --- | --- | --- | --- | --- | --- |
| 1 | PubMed | 1 | Pavel AB, et al. The proteomic skin profile of moderate-to-severe atopic dermatitis patients shows an inflammatory signature. 2020. PMID: 31669080. | No elderly-specific data | The available data were from middle-aged rather than older participants. |
| 2 | PubMed | 2 | Badi YE, et al. Mapping atopic dermatitis and anti-IL-22 response signatures to type 2-low severe neutrophilic asthma. 2022. PMID: 33891981. | No elderly-specific data | The adult sample was not stratified to provide an elderly subgroup. |
| 3 | PubMed | 3 | Wu Y, et al. Serum biomarker-based endotypes of atopic dermatitis in China and prediction for efficacy of dupilumab. 2023. PMID: 36756674. | No elderly-specific data | No elderly subgroup was identified; the original notes also indicate difficulty obtaining the full text. |
| 4 | PubMed | 4 | Caiazzo G, et al. Phenotype-endotype relationship in elderly atopic dermatitis and effects of dupilumab therapy: prospective study. 2025. PMID: 40095170. | No eligible/extractable biomarker-severity analysis | The study included older patients and follow-up data but no eligible severity-based analysis. |
| 5 | PubMed | 11 | Maruyama A, et al. Exploratory analyses of biomarkers in blood and stratum corneum in patients with atopic dermatitis. 2022. PMID: 36281133. | No eligible/extractable biomarker-severity analysis | Older participants were present, but no extractable elderly-specific effect data were reported. |
| 6 | PubMed | 12 | Elmelid A, et al. The Effect of Phototherapy on Systemic Inflammation Measured with Serum Vitamin D-Binding Protein and hsCRP in Patients with Inflammatory Skin Disease. 2024. PMID: 39201319. | No elderly-specific data | The study assessed AD and inflammatory markers but did not provide age-stratified data. |
| 7 | PubMed | 13 | Holm JG, et al. Immunoinflammatory Biomarkers in Serum Are Associated with Disease Severity in Atopic Dermatitis. 2021. PMID: 33730733. | No eligible/extractable biomarker-severity analysis | An older subgroup was suggested, but full-text or subgroup effect data were not available for extraction. |
| 8 | PubMed | 25 | Kou K, et al. Association of serum interleukin-18 and other biomarkers with disease severity in adults with atopic dermatitis. 2012. PMID: 22146836. | No elderly-specific data | No elderly subgroup data were reported. |
| 9 | PubMed | 34 | Sun R, et al. Observation of the efficacy of dupilumab for treatment of atopic dermatitis in the elderly. 2025. PMID: 40219560. | No eligible/extractable biomarker-severity analysis | The study included an elderly treatment sample but no baseline biomarker-severity analysis. |
| 10 | CNKI | 4 | Chen X. Role of senescent fibroblasts in the regulation of elderly atopic dermatitis [dissertation]. Peking Union Medical College, 2025. | No eligible/extractable biomarker-severity analysis | No eligible clinical severity measure was reported. |
| 11 | CNKI | 7 | Li L. Immune phenotypes of Chinese patients with atopic dermatitis and the mechanism of keratin 17-mediated mixed Th2/Th17 inflammation in elderly atopic dermatitis [dissertation]. Huazhong University of Science and Technology, 2024. | No eligible/extractable biomarker-severity analysis | Only summary statistics were available; the record was excluded at full-text screening. |
| 12 | CNKI | 10 | Tang Y. Clinical characteristics and related risk factors of elderly atopic-like dermatitis [dissertation]. Anhui Medical University, 2023. | No eligible/extractable biomarker-severity analysis | No relevant immune biomarker assessment was performed. |
| 13 | CNKI | 11 | Hao Y. Clinical characteristics and cytokine analysis of elderly atopic dermatitis [dissertation]. Henan University, 2022. | No eligible/extractable biomarker-severity analysis | Only summary statistics were available; the record was excluded at full-text screening. |

A total of 13 records were excluded after full-text assessment: 5 were excluded for lacking elderly-specific subgroup data (PubMed records 1, 2, 3, 12, and 25), and 8 were excluded for not reporting an eligible or extractable biomarker–severity correlation (PubMed records 4, 11, 13, and 34; CNKI records 4, 7, 10, and 11). The sum of these exclusions (5 + 8 = 13) matches the total number of full-text exclusions.

**1.5 Records of Two Reviewers Independently Screened**

| **Record ID (1–106)** | **Reviewer 1 Round 1 Screening** | **Reviewer 1 Round 2 Screening** | **Reviewer 2 Round 1 Screening** | **Reviewer 2 Round 2 Screening** |
| --- | --- | --- | --- | --- |
| 1 | Pass | Fail | Pass | Fail |
| 2 | Pass | Fail | Pass | Fail |
| 3 | Pass | Fail | Pass | Fail |
| 4 | Pass | Fail | Pass | Fail |
| 5 | Fail | Fail | Fail | Fail |
| 6 | Fail | Fail | Fail | Fail |
| 7 | Fail | Fail | Fail | Fail |
| 8 | Fail | Fail | Fail | Fail |
| 9 | Fail | Fail | Fail | Fail |
| 10 | Fail | Fail | Fail | Fail |
| 11 | Pass | Fail | Pass | Fail |
| 12 | Pass | Fail | Pass | Fail |
| 13 | Pass | Fail | Pass | Fail |
| 14 | Fail | Fail | Fail | Fail |
| 15 | Fail | Fail | Fail | Fail |
| 16 | Fail | Fail | Fail | Fail |
| 17 | Fail | Fail | Fail | Fail |
| 18 | Fail | Fail | Fail | Fail |
| 19 | Fail | Fail | Fail | Fail |
| 20 | Pass | Pass | Pass | Pass |
| 21 | Fail | Fail | Fail | Fail |
| 22 | Fail | Fail | Fail | Fail |
| 23 | Fail | Fail | Fail | Fail |
| 24 | Fail | Fail | Fail | Fail |
| 25 | Pass | Fail | Pass | Fail |
| 26 | Fail | Fail | Fail | Fail |
| 27 | Fail | Fail | Fail | Fail |
| 28 | Fail | Fail | Fail | Fail |
| 29 | Fail | Fail | Fail | Fail |
| 30 | Fail | Fail | Fail | Fail |
| 31 | Fail | Fail | Fail | Fail |
| 32 | Fail | Fail | Fail | Fail |
| 33 | Fail | Fail | Fail | Fail |
| 34 | Pass | Fail | Pass | Fail |
| 35 | Fail | Fail | Fail | Fail |
| 36 | Pass | Pass | Pass | Pass |
| 37 | Fail | Fail | Fail | Fail |
| 38 | Fail | Fail | Fail | Fail |
| 39 | Fail | Fail | Fail | Fail |
| 40 | Fail | Fail | Fail | Fail |
| 41 | Fail | Fail | Fail | Fail |
| 42 | Fail | Fail | Fail | Fail |
| 43 | Fail | Fail | Fail | Fail |
| 44 | Fail | Fail | Fail | Fail |
| 45 | Fail | Fail | Fail | Fail |
| 46 | Fail | Fail | Fail | Fail |
| 47 | Fail | Fail | Fail | Fail |
| 48 | Fail | Fail | Fail | Fail |
| 49 | Fail | Fail | Fail | Fail |
| 50 | Fail | Fail | Fail | Fail |
| 51 | Fail | Fail | Fail | Fail |
| 52 | Fail | Fail | Fail | Fail |
| 53 | Fail | Fail | Fail | Fail |
| 54 | Fail | Fail | Fail | Fail |
| 55 | Fail | Fail | Fail | Fail |
| 56 | Fail | Fail | Fail | Fail |
| 57 | Fail | Fail | Fail | Fail |
| 58 | Fail | Fail | Fail | Fail |
| 59 | Fail | Fail | Fail | Fail |
| 60 | Fail | Fail | Fail | Fail |
| 61 | Fail | Fail | Fail | Fail |
| 62 | Fail | Fail | Fail | Fail |
| 63 | Fail | Fail | Fail | Fail |
| 64 | Fail | Fail | Fail | Fail |
| 65 | Fail | Fail | Fail | Fail |
| 66 | Fail | Fail | Fail | Fail |
| 67 | Fail | Fail | Fail | Fail |
| 68 | Fail | Fail | Fail | Fail |
| 69 | Fail | Fail | Fail | Fail |
| 70 | Fail | Fail | Fail | Fail |
| 71 | Fail | Fail | Fail | Fail |
| 72 | Fail | Fail | Fail | Fail |
| 73 | Fail | Fail | Fail | Fail |
| 74 | Fail | Fail | Fail | Fail |
| 75 | Fail | Fail | Fail | Fail |
| 76 | Fail | Fail | Fail | Fail |
| 77 | Fail | Fail | Fail | Fail |
| 78 | Fail | Fail | Fail | Fail |
| 79 | Fail | Fail | Fail | Fail |
| 80 | Fail | Fail | Fail | Fail |
| 81 | Fail | Fail | Fail | Fail |
| 82 | Fail | Fail | Fail | Fail |
| 83 | Fail | Fail | Fail | Fail |
| 84 | Fail | Fail | Fail | Fail |
| 85 | Fail | Fail | Fail | Fail |
| 86 | Fail | Fail | Fail | Fail |
| 87 | Fail | Fail | Fail | Fail |
| 88 | Fail | Fail | Fail | Fail |
| 89 | Fail | Fail | Fail | Fail |
| 90 | Fail | Fail | Fail | Fail |
| 91 | Fail | Fail | Fail | Fail |
| 92 | Fail | Fail | Fail | Fail |
| 93 | Fail | Fail | Fail | Fail |
| 94 | Fail | Fail | Fail | Fail |
| 95 | Pass | Pass | Pass | Pass |
| 96 | Fail | Fail | Fail | Fail |
| 97 | Fail | Fail | Fail | Fail |
| 98 | Fail | Fail | Fail | Fail |
| 99 | Pass | Fail | Pass | Fail |
| 100 | Pass | Pass | Pass | Pass |
| 101 | Pass | Pass | Pass | Pass |
| 102 | Pass | Fail | Pass | Fail |
| 103 | Fail | Fail | Fail | Fail |
| 104 | Pass | Pass | Pass | Pass |
| 105 | Pass | Fail | Pass | Fail |
| 106 | Pass | Fail | Pass | Fail |

**1.6 Complete List of 106 Records Identified**

Records 1-95 were identified in PubMed and records 96-106 were identified in CNKI.

1. Pavel AB, Zhou L, Diaz A, Ungar B, Dan J, He H, Estrada YD, Xu H, Fernandes M, Renert-Yuval Y, Krueger JG, Guttman-Yassky E. The proteomic skin profile of moderate-to-severe atopic dermatitis patients shows an inflammatory signature. J Am Acad Dermatol. 2020 Mar;82(3):690-699. doi: 10.1016/j.jaad.2019.10.039. Epub 2019 Oct 25. PMID: 31669080.
2. Badi YE, Pavel AB, Pavlidis S, Riley JH, Bates S, Kermani NZ, Knowles R, Kolmert J, Wheelock CE, Worsley S, Uddin M, Alving K, Bakke PS, Behndig A, Caruso M, Chanez P, Fleming LJ, Fowler SJ, Frey U, Howarth P, Horváth I, Krug N, Maitland-van der Zee AH, Montuschi P, Roberts G, Sanak M, Shaw DE, Singer F, Sterk PJ, Djukanovic R, Dahlen SE, Guo YK, Chung KF, Guttman-Yassky E, Adcock IM; U-BIOPRED Study Group. Mapping atopic dermatitis and anti-IL-22 response signatures to type 2-low severe neutrophilic asthma. J Allergy Clin Immunol. 2022 Jan;149(1):89-101. doi: 10.1016/j.jaci.2021.04.010. Epub 2021 Apr 20. PMID: 33891981.
3. Wu Y, Gu C, Wang S, Yin H, Qiu Z, Luo Y, Li Z, Wang C, Yao X, Li W. Serum biomarker-based endotypes of atopic dermatitis in China and prediction for efficacy of dupilumab. Br J Dermatol. 2023 Apr 20;188(5):649-660. doi: 10.1093/bjd/ljad032. PMID: 36756674.
4. Caiazzo G, Napolitano M, Quaranta M, Picone V, Fabbrocini G, Patruno C. Phenotype-endotype relationship in elderly atopic dermatitis and effects of dupilumab therapy: prospective study. Arch Dermatol Res. 2025 Mar 17;317(1):575. doi: 10.1007/s00403-025-04090-5. PMID: 40095170.
5. Kosloski MP, Guttman-Yassky E, Cork MJ, Worm M, Nahm DH, Zhu X, Ruddy MK, Harel S, Kamal MA, Goulaouic H, Xu CR, Avetisova E, Davis JD, Nivens MC, Shabbir A, Radin A. Pharmacokinetics and pharmacodynamics of itepekimab in adults with moderate-to-severe atopic dermatitis: Results from two terminated phase II trials. Clin Transl Sci. 2024 Aug;17(8):e13874. doi: 10.1111/cts.13874. PMID: 39077906.
6. Simpson EL, Guttman-Yassky E, Pawlikowski J, Ghorayeb EG, Ota T, Lebwohl MG. Interleukin-1alpha inhibitor bermekimab in patients with atopic dermatitis: randomized and nonrandomized studies. Arch Dermatol Res. 2024 Aug 30;316(8):589. doi: 10.1007/s00403-024-03319-z. PMID: 39214920.
7. Fehr D, Huynh-Tran VH, Maintz L, Niederseer D, Ameri M, Dreher A, Study Group CC, Akdis CA, Lauener R, Rhyner C, Traidl-Hoffmann C, Schmid-Grendelmeier P, Bieber T, Brüggen MC. Deciphering the Connection Between Atopic Dermatitis and Cardiovascular Diseases: Analysis of Clinical Associations and Cardiometabolic Proteins. Allergy. 2025 Aug;80(8):2187-2200. doi: 10.1111/all.16588. Epub 2025 May 19. PMID: 40386898.
8. Chopra R, Vakharia PP, Sacotte R, Patel N, Immaneni S, White T, Kantor R, Hsu DY, Silverberg JI. Relationship between EASI and SCORAD severity assessments for atopic dermatitis. J Allergy Clin Immunol. 2017 Dec;140(6):1708-1710.e1. doi: 10.1016/j.jaci.2017.04.052. Epub 2017 Jun 15. PMID: 28625808.
9. He H, Del Duca E, Diaz A, Kim HJ, Gay-Mimbrera J, Zhang N, Wu J, Beaziz J, Estrada Y, Krueger JG, Pavel AB, Ruano J, Guttman-Yassky E. Mild atopic dermatitis lacks systemic inflammation and shows reduced nonlesional skin abnormalities. J Allergy Clin Immunol. 2021 Apr;147(4):1369-1380. doi: 10.1016/j.jaci.2020.08.041. Epub 2020 Oct 1. PMID: 33011244.
10. Xu M, Deng H, Zhang X, Deng J, Yu W, Han L, Yan Y, Yao D, Yu J, Ye S, Cui J, Hu D, Jia Y, Dong Z, Xu D, Yu X, Lu C. Systematic analysis of serum peptidase inhibitor 3 in psoriasis diagnosis and treatment. Clin Rheumatol. 2024 Nov;43(11):3361-3372. doi: 10.1007/s10067-024-07138-5. Epub 2024 Sep 17. PMID: 39287701.
11. Maruyama A, Tamagawa-Mineoka R, Nishigaki H, Masuda K, Katoh N. Exploratory analyses of biomarkers in blood and stratum corneum in patients with atopic dermatitis. Medicine (Baltimore). 2022 Oct 21;101(42):e31267. doi: 10.1097/MD.0000000000031267. PMID: 36281133.
12. Elmelid A, Vandikas MS, Gillstedt M, Alsterholm M, Osmancevic A. The Effect of Phototherapy on Systemic Inflammation Measured with Serum Vitamin D-Binding Protein and hsCRP in Patients with Inflammatory Skin Disease. Int J Mol Sci. 2024 Aug 8;25(16):8632. doi: 10.3390/ijms25168632. PMID: 39201319.
13. Holm JG, Hurault G, Agner T, Clausen ML, Kezic S, Tanaka RJ, Thomsen SF. Immunoinflammatory Biomarkers in Serum Are Associated with Disease Severity in Atopic Dermatitis. Dermatology. 2021;237(4):513-520. doi: 10.1159/000514503. Epub 2021 Mar 17. PMID: 33730733.
14. Ban GY, Kim MY, Yoo HS, Nahm DH, Ye YM, Shin YS, Park HS. Clinical features of elderly chronic urticaria. Korean J Intern Med. 2014 Nov;29(6):800-6. doi: 10.3904/kjim.2014.29.6.800. Epub 2014 Oct 31. PMID: 25378979.
15. Ungar B, Garcet S, Gonzalez J, Dhingra N, Correa da Rosa J, Shemer A, Krueger JG, Suarez-Farinas M, Guttman-Yassky E. An Integrated Model of Atopic Dermatitis Biomarkers Highlights the Systemic Nature of the Disease. J Invest Dermatol. 2017 Mar;137(3):603-613. doi: 10.1016/j.jid.2016.09.037. Epub 2016 Nov 4. PMID: 27825969.
16. Quaade AS, Wang X, Sølberg JBK, McCauley BD, Thyssen JP, Becker C, Johansen JD. Inflammatory plasma signature of chronic hand eczema: Associations with aetiological and clinical subtypes. J Eur Acad Dermatol Venereol. 2024 Jun;38(6):1101-1111. doi: 10.1111/jdv.19742. Epub 2023 Dec 27. PMID: 38151335.
17. Droitcourt C, Vittrup I, Kerbrat S, Egeberg A, Thyssen JP. Risk of systemic infections in adults with atopic dermatitis: A nationwide cohort study. J Am Acad Dermatol. 2021 Feb;84(2):290-299. doi: 10.1016/j.jaad.2020.07.111. Epub 2020 Aug 1. PMID: 32750384.
18. Orfali RL, Shimizu MM, Takaoka R, Zaniboni MC, Ishizaki AS, Costa AA, Tiba AP, Sato MN, Aoki V. Atopic dermatitis in adults: clinical and epidemiological considerations. Rev Assoc Med Bras (1992). 2013 May-Jun;59(3):270-5. doi: 10.1016/j.ramb.2012.12.004. Epub 2013 May 13. PMID: 23680276.
19. Song HS, Jung SE, Kim YC, Lee ES. Nipple eczema, an indicative manifestation of atopic dermatitis? A clinical, histological, and immunohistochemical study. Am J Dermatopathol. 2015 Apr;37(4):284-8. doi: 10.1097/DAD.0000000000000195. PMID: 25079201.
20. He H, Li R, Choi S, Zhou L, Pavel A, Estrada YD, Krueger JG, Guttman-Yassky E. Increased cardiovascular and atherosclerosis markers in blood of older patients with atopic dermatitis. Ann Allergy Asthma Immunol. 2020 Jan;124(1):70-78. doi: 10.1016/j.anai.2019.10.013. Epub 2019 Oct 14. PMID: 31622668.
21. Ronceray S, Benkalfate L, Saillard C, Ezzedine K, Adamski H, Dupuy A, Droitcourt C. [Atopic dermatitis severity and vitamin D concentration: a cross-sectional study]. Ann Dermatol Venereol. 2014 Apr;141(4):265-71. doi: 10.1016/j.annder.2014.01.013. Epub 2014 Feb 24. PMID: 24703640.
22. Beck LA, Boguniewicz M, Hata T, Schneider LC, Hanifin J, Gallo R, Paller AS, Lieff S, Reese J, Zaccaro D, Milgrom H, Barnes KC, Leung DY. Phenotype of atopic dermatitis subjects with a history of eczema herpeticum. J Allergy Clin Immunol. 2009 Aug;124(2):260-9, 269.e1-7. doi: 10.1016/j.jaci.2009.05.020. Epub 2009 Jun 27. PMID: 19541356.
23. Umeda M, Origuchi T, Kawashiri SY, Koga T, Ichinose K, Furukawa K, Sato T, Tsuji S, Endo Y, Takatani A, Shimizu T, Fukui S, Iwamoto N, Igawa T, Tamai M, Nakamura H, Kawakami A. Thymus and Activation-regulated Chemokine as a Biomarker for IgG4-related Disease. Sci Rep. 2020 Apr 7;10(1):6010. doi: 10.1038/s41598-020-62941-9. PMID: 32265499.
24. Su H, Luo Y, Sun J, Liu X, Ling S, Xu B, Zhang Y, Liu J, Li W, Wang B, Yao X. Transglutaminase 3 Promotes Skin Inflammation in Atopic Dermatitis by Activating Monocyte-Derived Dendritic Cells via DC-SIGN. J Invest Dermatol. 2020 Feb;140(2):370-379.e8. doi: 10.1016/j.jid.2019.07.703. Epub 2019 Aug 16. PMID: 31425706.
25. Kou K, Aihara M, Matsunaga T, Chen H, Taguri M, Morita S, Fujita H, Yamaguchi Y, Kambara T, Ikezawa Z. Association of serum interleukin-18 and other biomarkers with disease severity in adults with atopic dermatitis. Arch Dermatol Res. 2012 May;304(4):305-12. doi: 10.1007/s00403-011-1198-9. Epub 2011 Dec 7. PMID: 22146836.
26. Jaworek AK, Szafraniec K, Zuber Z, Wojas-Pelc A, Jaworek J. Interleukin 25, thymic stromal lymphopoietin and house dust mites in pathogenesis of atopic dermatitis. J Physiol Pharmacol. 2020 Apr;71(2). doi: 10.26402/jpp.2020.2.14. Epub 2020 Aug 8. PMID: 32776912.
27. Martinez-Escala ME, Posligua AL, Wickless H, Rutherford A, Sable KA, Rubio-Gonzalez B, Zhou XA, Kaplan JB, Pro B, Choi J, Querfeld C, Rosen ST, Guitart J. Progression of undiagnosed cutaneous lymphoma after anti-tumor necrosis factor-alpha therapy. J Am Acad Dermatol. 2018 Jun;78(6):1068-1076. doi: 10.1016/j.jaad.2017.12.068. Epub 2018 Jan 4. PMID: 29307643.
28. Khattri S, Shemer A, Rozenblit M, Dhingra N, Czarnowicki T, Finney R, Gilleaudeau P, Sullivan-Whalen M, Zheng X, Xu H, Cardinale I, de Guzman Strong C, Gonzalez J, Suárez-Fariñas M, Krueger JG, Guttman-Yassky E. Cyclosporine in patients with atopic dermatitis modulates activated inflammatory pathways and reverses epidermal pathology. J Allergy Clin Immunol. 2014 Jun;133(6):1626-34. doi: 10.1016/j.jaci.2014.03.003. Epub 2014 Apr 29. PMID: 24786238.
29. Meyersburg D, Laimer M, Kugler A, Mühlthaler E, Lindlbauer N, Hitzl W, Rohde E, Bauer JW, Grabmer C. Single-use IgE-selective immunoadsorber column for the treatment of severe atopic dermatitis. J Clin Apher. 2020 Jan;35(1):50-58. doi: 10.1002/jca.21759. Epub 2019 Nov 22. PMID: 31755575.
30. Yamamoto K, Yokoyama K, Matsukawa T, Kato S, Kato S, Yamada K, Hirota T. Efficacy of prolonged ingestion of Lactobacillus acidophilus L-92 in adult patients with atopic dermatitis. J Dairy Sci. 2016 Jul;99(7):5039-5046. doi: 10.3168/jds.2015-10605. Epub 2016 Apr 20. PMID: 27108169.
31. Suga H, Sugaya M, Miyagaki T, Kawaguchi M, Morimura S, Kai H, Kagami S, Ohmatsu H, Fujita H, Asano Y, Tada Y, Kadono T, Sato S. Serum visfatin levels in patients with atopic dermatitis and cutaneous T-cell lymphoma. Eur J Dermatol. 2013 Sep-Oct;23(5):629-35. doi: 10.1684/ejd.2013.2107. PMID: 24001451.
32. Oláh A, Szabó-Papp J, Soeberdt M, Knie U, Dähnhardt-Pfeiffer S, Abels C, Bíró T. Echinacea purpurea-derived alkylamides exhibit potent anti-inflammatory effects and alleviate clinical symptoms of atopic eczema. J Dermatol Sci. 2017 Oct;88(1):67-77. doi: 10.1016/j.jdermsci.2017.05.015. Epub 2017 May 27. PMID: 28610718.
33. Mittermann I, Wikberg G, Johansson C, Lupinek C, Lundeberg L, Crameri R, Valenta R, Scheynius A. IgE Sensitization Profiles Differ between Adult Patients with Severe and Moderate Atopic Dermatitis. PLoS One. 2016 May 26;11(5):e0156077. doi: 10.1371/journal.pone.0156077. eCollection 2016. PMID: 27228091.
34. Sun R, Wu Y, DI M, Wang X. [Observation of the efficacy of dupilumab for treatment of atopic dermatitis in the elderly]. Beijing Da Xue Xue Bao Yi Xue Ban. 2025 Apr 18;57(2):298-302. doi: 10.19723/j.issn.1671-167X.2025.02.012. PMID: 40219560.
35. Zink A, Gensbaur A, Zirbs M, Seifert F, Suarez IL, Mourantchanian V, Weidinger S, Mempel M, Ring J, Ollert M. Targeting IgE in Severe Atopic Dermatitis with a Combination of Immunoadsorption and Omalizumab. Acta Derm Venereol. 2016 Jan;96(1):72-6. doi: 10.2340/00015555-2165. PMID: 26059424.
36. Zhou L, Leonard A, Pavel AB, Malik K, Raja A, Glickman J, Estrada YD, Peng X, Del Duca E, Sanz-Cabanillas J, Ruano J, Xu H, Zhang N, Wen HC, Gonzalez J, Garcet S, Krueger JG, Guttman-Yassky E. Age-specific changes in the molecular phenotype of patients with moderate-to-severe atopic dermatitis. J Allergy Clin Immunol. 2019 Jul;144(1):144-156. doi: 10.1016/j.jaci.2019.01.015. Epub 2019 Jan 24. PMID: 30685456.
37. Aizawa N, Ishiuji Y, Tominaga M, Sakata S, Takahashi N, Yanaba K, Umezawa Y, Asahina A, Kimura U, Suga Y, Takamori K, Nakagawa H. Relationship between the Degrees of Itch and Serum Lipocalin-2 Levels in Patients with Psoriasis. J Immunol Res. 2019 Jan 21;2019:8171373. doi: 10.1155/2019/8171373. eCollection 2019. PMID: 30805373.
38. Czarnowicki T, He H, Leonard A, Kim HJ, Kameyama N, Pavel AB, Li R, Estrada Y, Wen HC, Kimmel GW, Kim HJ, Chima M, Lebwohl M, Krueger JG, Guttman-Yassky E. Blood endotyping distinguishes the profile of vitiligo from that of other inflammatory and autoimmune skin diseases. J Allergy Clin Immunol. 2019 Jun;143(6):2095-2107. doi: 10.1016/j.jaci.2018.11.031. Epub 2018 Dec 18. PMID: 30576756.
39. Sanyal RD, Pavel AB, Glickman J, Chan TC, Zheng X, Zhang N, Cueto I, Peng X, Estrada Y, Fuentes-Duculan J, Alexis AF, Krueger JG, Guttman-Yassky E. Atopic dermatitis in African American patients is T(H)2/T(H)22-skewed with T(H)1/T(H)17 attenuation. Ann Allergy Asthma Immunol. 2019 Jan;122(1):99-110.e6. doi: 10.1016/j.anai.2018.08.024. Epub 2018 Sep 14. PMID: 30223113.
40. Noda S, Suárez-Fariñas M, Ungar B, Kim SJ, de Guzman Strong C, Xu H, Peng X, Estrada YD, Nakajima S, Honda T, Shin JU, Lee H, Krueger JG, Lee KH, Kabashima K, Guttman-Yassky E. The Asian atopic dermatitis phenotype combines features of atopic dermatitis and psoriasis with increased TH17 polarization. J Allergy Clin Immunol. 2015 Nov;136(5):1254-64. doi: 10.1016/j.jaci.2015.08.015. Epub 2015 Oct 1. PMID: 26428954.
41. Silverberg JI, Guttman-Yassky E, Gooderham M, Worm M, Rippon S, O'Quinn S, van der Merwe R, Kragh N, Kurbasic A, Wollenberg A. Health-related quality of life with tralokinumab in moderate-to-severe atopic dermatitis: A phase 2b randomized study. Ann Allergy Asthma Immunol. 2021 May;126(5):576-583.e4. doi: 10.1016/j.anai.2020.12.004. Epub 2020 Dec 15. PMID: 33333295.
42. Chiba T, Tatematsu S, Nakao M, Furue M. Urinary biopyrrin: a potential inflammatory marker of atopic dermatitis. Ann Allergy Asthma Immunol. 2014 Feb;112(2):182-3. doi: 10.1016/j.anai.2013.12.011. PMID: 24468264.
43. Otobe S, Sugaya M, Nakajima R, Oka T, Takahashi N, Kabasawa M, Miyagaki T, Asano Y, Sato S. Increased interleukin-36gamma expression in skin and sera of patients with atopic dermatitis and mycosis fungoides/Sezary syndrome. J Dermatol. 2018 Apr;45(4):468-471. doi: 10.1111/1346-8138.14198. Epub 2018 Jan 3. PMID: 29314201.
44. Inui S, Noguchi F, Nakajima T, Itami S. Serum thymus and activation-regulated chemokine as disease activity and response biomarker in alopecia areata. J Dermatol. 2013 Nov;40(11):881-5. doi: 10.1111/1346-8138.12273. Epub 2013 Sep 17. PMID: 24102731.
45. Tremp M, Knafla I, Burg G, Wüthrich B, Schmid-Grendelmeier P. 'EASIdig'--a digital tool to document disease activity in atopic dermatitis. Dermatology. 2011;223(1):68-73. doi: 10.1159/000330333. Epub 2011 Aug 25. PMID: 21865675.
46. Peng WM, Jenneck C, Bussmann C, Bogdanow M, Hart J, Leung DY, Bieber T, Eis-Hübinger AM, Novak N. Risk factors of atopic dermatitis patients for eczema herpeticum. J Invest Dermatol. 2007 May;127(5):1261-3. doi: 10.1038/sj.jid.5700657. Epub 2006 Dec 14. PMID: 17170734.
47. Ohta S, Okamoto M, Fujimoto K, Sakamoto N, Takahashi K, Yamamoto H, Kushima H, Ishii H, Akasaka K, Ono J, Kamei A, Azuma Y, Matsumoto H, Yamaguchi Y, Aihara M, Johkoh T, Kawaguchi A, Ichiki M, Sagara H, Kadota JI, Hanaoka M, Hayashi SI, Kohno S, Hoshino T, Izuhara K; Consortium for Development of Diagnostics for Pulmonary Fibrosis Patients (CoDD-PF). The usefulness of monomeric periostin as a biomarker for idiopathic pulmonary fibrosis. PLoS One. 2017 Mar 29;12(3):e0174547. doi: 10.1371/journal.pone.0174547. eCollection 2017. PMID: 28355256.
48. Czarnowicki T, Gonzalez J, Bonifacio KM, Shemer A, Xiangyu P, Kunjravia N, Malajian D, Fuentes-Duculan J, Esaki H, Noda S, Estrada Y, Xu H, Zheng X, Krueger JG, Guttman-Yassky E. Diverse activation and differentiation of multiple B-cell subsets in patients with atopic dermatitis but not in patients with psoriasis. J Allergy Clin Immunol. 2016 Jan;137(1):118-129.e5. doi: 10.1016/j.jaci.2015.08.027. Epub 2015 Oct 5. PMID: 26441226.
49. Ilves T, Harvima IT. OX40 ligand and OX40 are increased in atopic dermatitis lesions but do not correlate with clinical severity. J Eur Acad Dermatol Venereol. 2013 Feb;27(2):e197-205. doi: 10.1111/j.1468-3083.2012.04587.x. Epub 2012 May 31. PMID: 22646697.
50. Czarnowicki T, Malajian D, Khattri S, Correa da Rosa J, Dutt R, Finney R, Dhingra N, Xiangyu P, Xu H, Estrada YD, Zheng X, Gilleaudeau P, Sullivan-Whalen M, Suaréz-Fariñas M, Shemer A, Krueger JG, Guttman-Yassky E. Petrolatum: Barrier repair and antimicrobial responses underlying this "inert" moisturizer. J Allergy Clin Immunol. 2016 Apr;137(4):1091-1102.e7. doi: 10.1016/j.jaci.2015.08.013. Epub 2015 Oct 1. PMID: 26431582.
51. Clausen ML, Jungersted JM, Andersen PS, Slotved HC, Krogfelt KA, Agner T. Human beta-defensin-2 as a marker for disease severity and skin barrier properties in atopic dermatitis. Br J Dermatol. 2013 Sep;169(3):587-93. doi: 10.1111/bjd.12419. PMID: 23647067.
52. Suárez-Fariñas M, Dhingra N, Gittler J, Shemer A, Cardinale I, de Guzman Strong C, Krueger JG, Guttman-Yassky E. Intrinsic atopic dermatitis shows similar TH2 and higher TH17 immune activation compared with extrinsic atopic dermatitis. J Allergy Clin Immunol. 2013 Aug;132(2):361-70. doi: 10.1016/j.jaci.2013.04.046. Epub 2013 Jun 15. PMID: 23777851.
53. Czarnowicki T, Gonzalez J, Shemer A, Malajian D, Xu H, Zheng X, Khattri S, Gilleaudeau P, Sullivan-Whalen M, Suárez-Fariñas M, Krueger JG, Guttman-Yassky E. Severe atopic dermatitis is characterized by selective expansion of circulating TH2/TC2 and TH22/TC22, but not TH17/TC17, cells within the skin-homing T-cell population. J Allergy Clin Immunol. 2015 Jul;136(1):104-115.e7. doi: 10.1016/j.jaci.2015.01.020. Epub 2015 Mar 3. PMID: 25748064.
54. Kanapathy M, Simpson R, Madden L, Thrasivoulou C, Mosahebi A, Becker DL, Richards T. Upregulation of epidermal gap junctional proteins in patients with venous disease. Br J Surg. 2018 Jan;105(1):59-67. doi: 10.1002/bjs.10653. Epub 2017 Nov 16. PMID: 29143961.
55. Bengtsson A, Holm L, Bäck O, Fransson J, Scheynius A. Elevated serum levels of soluble CD30 in patients with atopic dermatitis (AD). Clin Exp Immunol. 1997 Sep;109(3):533-7. doi: 10.1046/j.1365-2249.1997.4731373.x. PMID: 9328133.
56. Emtestam L, Hagströmer L, Dou YC, Sartorius K, Johansson O. PGP 9.5 distribution patterns in biopsies from early lesions of atopic dermatitis. Arch Dermatol Res. 2012 Dec;304(10):781-5. doi: 10.1007/s00403-012-1246-0. Epub 2012 Jun 2. PMID: 22660851.
57. Wu J, Zhang K, Liao Y, Yang C, Ge J, Wu C, Yu B, Zhong W, Shao Y, Dou X. Serum SERPINB3/4 in Moderate-to-Severe Prurigo Nodularis: A Potential Biomarker for Disease Severity and Eosinophil-Related Parameters. Immun Inflamm Dis. 2025 Sep;13(9):e70263. doi: 10.1002/iid3.70263. PMID: 40995892.
58. Yatagai T, Shimauchi T, Yamaguchi H, Sakabe JI, Aoshima M, Ikeya S, Tatsuno K, Fujiyama T, Ito T, Ojima T, Tokura Y. Sensitive skin is highly frequent in extrinsic atopic dermatitis and correlates with disease severity markers but not necessarily with skin barrier impairment. J Dermatol Sci. 2018 Jan;89(1):33-39. doi: 10.1016/j.jdermsci.2017.10.011. Epub 2017 Nov 2. PMID: 29122406.
59. Bussmann C, Maintz L, Hart J, Allam JP, Vrtala S, Chen KW, Bieber T, Thomas WR, Valenta R, Zuberbier T, Sager A, Novak N. Clinical improvement and immunological changes in atopic dermatitis patients undergoing subcutaneous immunotherapy with a house dust mite allergoid: a pilot study. Clin Exp Allergy. 2007 Sep;37(9):1277-85. doi: 10.1111/j.1365-2222.2007.02783.x. PMID: 17845407.
60. Tofte SJ, Papp K, Sadick N, Bohnert K, Simpson E, Thaçi D, Bieber T, Blauvelt A, Sofen H, Gooderham M, Chen Z, Gadkari A, Eckert L, Graham NMH, Pirozzi G, Ardeleanu M. Efficacy and safety of dupilumab for the treatment of moderate-to-severe atopic dermatitis in adults: A pooled analysis of two phase 2 clinical trials. J Am Assoc Nurse Pract. 2018 Sep;30(9):529-541. doi: 10.1097/JXX.0000000000000088. PMID: 30211823.
61. Nakayama T, Morimoto K, Uchiyama K, Kusahana E, Washida N, Azegami T, Kanda T, Yoshida T, Itoh H. Serum thymus and activation-regulated chemokine level is associated with the severity of chronic kidney disease-associated pruritus in patients undergoing peritoneal dialysis. Perit Dial Int. 2022 Jul;42(4):415-424. doi: 10.1177/08968608221085432. Epub 2022 Mar 16. PMID: 35296169.
62. Batista DI, Perez L, Orfali RL, Zaniboni MC, Samorano LP, Pereira NV, Sotto MN, Ishizaki AS, Oliveira LM, Sato MN, Aoki V. Profile of skin barrier proteins (filaggrin, claudins 1 and 4) and Th1/Th2/Th17 cytokines in adults with atopic dermatitis. J Eur Acad Dermatol Venereol. 2015 Jun;29(6):1091-5. doi: 10.1111/jdv.12753. Epub 2014 Oct 1. PMID: 25271795.
63. Sonesson A, Bartosik J, Christiansen J, Roscher I, Nilsson F, Schmidtchen A, Bäck O. Sensitization to skin-associated microorganisms in adult patients with atopic dermatitis is of importance for disease severity. Acta Derm Venereol. 2013 May;93(3):340-5. doi: 10.2340/00015555-1465. PMID: 23073977.
64. Katagiri K, Arakawa S, Hatano Y. In vivo levels of IL-4, IL-10, TGF-beta1 and IFN-gamma mRNA of the peripheral blood mononuclear cells in patients with alopecia areata in comparison to those in patients with atopic dermatitis. Arch Dermatol Res. 2007 Jan;298(8):397-401. doi: 10.1007/s00403-006-0700-2. Epub 2006 Sep 22. PMID: 17021766.
65. Song TW, Sohn MH, Kim ES, Kim KW, Kim KE. Increased serum thymus and activation-regulated chemokine and cutaneous T cell-attracting chemokine levels in children with atopic dermatitis. Clin Exp Allergy. 2006 Mar;36(3):346-51. doi: 10.1111/j.1365-2222.2006.02430.x. PMID: 16499646.
66. Hayashida S, Uchi H, Moroi Y, Furue M. Decrease in circulating Th17 cells correlates with increased levels of CCL17, IgE and eosinophils in atopic dermatitis. J Dermatol Sci. 2011 Mar;61(3):180-6. doi: 10.1016/j.jdermsci.2010.10.013. Epub 2010 Nov 3. PMID: 21255981.
67. Miyagaki T, Sugaya M, Suga H, Ohmatsu H, Fujita H, Asano Y, Tada Y, Kadono T, Sato S. Increased CCL18 expression in patients with cutaneous T-cell lymphoma: association with disease severity and prognosis. J Eur Acad Dermatol Venereol. 2013 Jan;27(1):e60-7. doi: 10.1111/j.1468-3083.2012.04495.x. Epub 2012 Mar 9. PMID: 22404649.
68. Gao L, Bin L, Rafaels NM, Huang L, Potee J, Ruczinski I, Beaty TH, Paller AS, Schneider LC, Gallo R, Hanifin JM, Beck LA, Geha RS, Mathias RA, Barnes KC, Leung DYM. Targeted deep sequencing identifies rare loss-of-function variants in IFNGR1 for risk of atopic dermatitis complicated by eczema herpeticum. J Allergy Clin Immunol. 2015 Dec;136(6):1591-1600. doi: 10.1016/j.jaci.2015.06.047. Epub 2015 Sep 3. PMID: 26343451.
69. Uyl-de Groot CA, Löwenberg B, Vellenga E, Suciu S, Willemze R, Rutten FF. Cost-effectiveness and quality-of-life assessment of GM-CSF as an adjunct to intensive remission induction chemotherapy in elderly patients with acute myeloid leukemia. Br J Haematol. 1998 Mar;100(4):629-36. doi: 10.1046/j.1365-2141.1998.00635.x. PMID: 9531327.
70. Ilves T, Tiitu V, Suttle MM, Saarinen JV, Harvima IT. Epidermal Expression of Filaggrin/Profilaggrin Is Decreased in Atopic Dermatitis: Reverse Association With Mast Cell Tryptase and IL-6 but Not With Clinical Severity. Dermatitis. 2015 Nov-Dec;26(6):260-7. doi: 10.1097/DER.0000000000000143. PMID: 26551604.
71. Schulte-Herbrüggen O, Fölster-Holst R, von Elstermann M, Augustin M, Hellweg R. Clinical relevance of nerve growth factor serum levels in patients with atopic dermatitis and psoriasis. Int Arch Allergy Immunol. 2007;144(3):211-6. doi: 10.1159/000103994. Epub 2007 Jun 18. PMID: 17579279.
72. Hanifin JM, Schneider LC, Leung DY, Ellis CN, Jaffe HS, Izu AE, Bucalo LR, Hirabayashi SE, Tofte SJ, Cantu-Gonzales G, et al. Recombinant interferon gamma therapy for atopic dermatitis. J Am Acad Dermatol. 1993 Feb;28(2 Pt 1):189-97. doi: 10.1016/0190-9622(93)70026-p. PMID: 8432915.
73. Marzano AV, Tedeschi A, Berti E, Fanoni D, Crosti C, Cugno M. Activation of coagulation in bullous pemphigoid and other eosinophil-related inflammatory skin diseases. Clin Exp Immunol. 2011 Jul;165(1):44-50. doi: 10.1111/j.1365-2249.2011.04391.x. Epub 2011 Apr 13. PMID: 21488867.
74. Kiiski V, Karlsson O, Remitz A, Reitamo S. High serum total IgE predicts poor long-term outcome in atopic dermatitis. Acta Derm Venereol. 2015 Nov;95(8):943-7. doi: 10.2340/00015555-2126. PMID: 25916555.
75. Orfali RL, Sato MN, Takaoka R, Azor MH, Rivitti EA, Hanifin JM, Aoki V. Atopic dermatitis in adults: evaluation of peripheral blood mononuclear cells proliferation response to Staphylococcus aureus enterotoxins A and B and analysis of interleukin-18 secretion. Exp Dermatol. 2009 Jul;18(7):628-33. doi: 10.1111/j.1600-0625.2009.00842.x. Epub 2009 Mar 17. PMID: 19469892.
76. Sugaya M, Miyagaki T, Ohmatsu H, Suga H, Kai H, Kamata M, Fujita H, Asano Y, Tada Y, Kadono T, Okochi H, Sato S. Association of the numbers of CD163(+) cells in lesional skin and serum levels of soluble CD163 with disease progression of cutaneous T cell lymphoma. J Dermatol Sci. 2012 Oct;68(1):45-51. doi: 10.1016/j.jdermsci.2012.07.007. Epub 2012 Jul 27. PMID: 22884782.
77. Paller AS, Renert-Yuval Y, Suprun M, Esaki H, Oliva M, Huynh TN, Ungar B, Kunjravia N, Friedland R, Peng X, Zheng X, Estrada YD, Krueger JG, Choate KA, Suárez-Fariñas M, Guttman-Yassky E. An IL-17-dominant immune profile is shared across the major orphan forms of ichthyosis. J Allergy Clin Immunol. 2017 Jan;139(1):152-165. doi: 10.1016/j.jaci.2016.07.019. Epub 2016 Aug 20. PMID: 27554821.
78. Song Y, Shi YH, He C, Liu CQ, Wang JS, Zhao YJ, Guo YM, Wu RJ, Feng XY, Liu ZJ. Severe Henoch-Schönlein purpura with infliximab for ulcerative colitis. World J Gastroenterol. 2015 May 21;21(19):6082-7. doi: 10.3748/wjg.v21.i19.6082. PMID: 26019477.
79. Siebra MX, Santos MA, Almeida TL, Leite AC, Cunha FQ, Rocha FA. Evidence for the participation of nitric oxide in pemphigus. Braz J Med Biol Res. 2006 May;39(5):671-5. doi: 10.1590/s0100-879x2006000500014. Epub 2006 Apr 20. PMID: 16648905.
80. López-Lerma I, Estrach MT. A distinct profile of serum levels of soluble intercellular adhesion molecule-1 and intercellular adhesion molecule-3 in mycosis fungoides and Sézary syndrome. J Am Acad Dermatol. 2009 Aug;61(2):263-70. doi: 10.1016/j.jaad.2009.03.041. PMID: 19615537.
81. Katagiri K, Itami S, Hatano Y, Takayasu S. Increased levels of IL-13 mRNA, but not IL-4 mRNA, are found in vivo in peripheral blood mononuclear cells (PBMC) of patients with atopic dermatitis (AD). Clin Exp Immunol. 1997 May;108(2):289-94. doi: 10.1046/j.1365-2249.1997.d01-1015.x. PMID: 9158100.
82. Yanaba K, Yoshizaki A, Asano Y, Kadono T, Sato S. Serum interleukin 9 levels are increased in patients with systemic sclerosis: association with lower frequency and severity of pulmonary fibrosis. J Rheumatol. 2011 Oct;38(10):2193-7. doi: 10.3899/jrheum.110268. Epub 2011 Aug 1. PMID: 21807780.
83. Källström E, Roscher I, Andreasson A, Bäck O, van Hage-Hamsten M. Decreased frequency of intracellular IFN-gamma producing T cells in whole blood preparations from patients with atopic dermatitis. Exp Dermatol. 2002 Dec;11(6):556-63. doi: 10.1034/j.1600-0625.2002.110608.x. PMID: 12473063.
84. Miranda E, Roberts J, Novick S, Lapointe JM, Bruijnzeel-Koomen C, Thijs J, Sleeman MA, May RD, Hijnen D, Strickland I. Immunohistochemical Characterization of the IL-13:IL-4 Receptor alpha Axis in the Skin of Adult Patients with Moderate to Severe Atopic Dermatitis and Healthy Controls. J Invest Dermatol. 2021 Feb;141(2):440-443.e4. doi: 10.1016/j.jid.2020.05.108. Epub 2020 Jun 27. PMID: 32598926.
85. Vasiadi M, Therianou A, Sideri K, Smyrnioti M, Sismanopoulos N, Delivanis DA, Asadi S, Katsarou-Katsari A, Petrakopoulou T, Theoharides A, Antoniou C, Papadavid E, Stavrianeas N, Kalogeromitros D, Theoharides TC. Increased serum CRH levels with decreased skin CRHR-1 gene expression in psoriasis and atopic dermatitis. J Allergy Clin Immunol. 2012 May;129(5):1410-3. doi: 10.1016/j.jaci.2012.01.041. Epub 2012 Feb 22. PMID: 22360979.
86. van Velsen SG, Haeck IM, Bruijnzeel-Koomen CA, de Bruin-Weller MS. First experience with enteric-coated mycophenolate sodium (Myfortic) in severe recalcitrant adult atopic dermatitis: an open label study. Br J Dermatol. 2009 Mar;160(3):687-91. doi: 10.1111/j.1365-2133.2008.08978.x. Epub 2008 Dec 11. PMID: 19120337.
87. Ohmatsu H, Sugaya M, Miyagaki T, Suga H, Fujita H, Asano Y, Tada Y, Kadono T, Sato S. BAFF levels are increased in lesional skin and sera in patients with cutaneous T-cell lymphoma. Br J Dermatol. 2012 Aug;167(2):359-67. doi: 10.1111/j.1365-2133.2012.10998.x. Epub 2012 Jul 5. PMID: 22512719.
88. Hirao H, Jinnin M, Ichihara A, Fujisawa A, Makino K, Kajihara I, Sakai K, Fukushima S, Inoue Y, Ihn H. Detection of hair root miR-19a as a novel diagnostic marker for psoriasis. Eur J Dermatol. 2013 Nov-Dec;23(6):807-11. doi: 10.1684/ejd.2013.2190. PMID: 24192448.
89. Mrabet-Dahbi S, Breuer K, Klotz M, Herz U, Heeg K, Werfel T, Renz H. Deficiency in immunoglobulin G2 antibodies against staphylococcal enterotoxin C1 defines a subgroup of patients with atopic dermatitis. Clin Exp Allergy. 2005 Mar;35(3):274-81. doi: 10.1111/j.1365-2222.2005.02192.x. PMID: 15784103.
90. Peschen M, Grenz H, Brand-Saberi B, Bunaes M, Simon JC, Schöpf E, Vanscheidt W. Increased expression of platelet-derived growth factor receptor alpha and beta and vascular endothelial growth factor in the skin of patients with chronic venous insufficiency. Arch Dermatol Res. 1998 Jun;290(6):291-7. doi: 10.1007/s004030050307. PMID: 9705159.
91. Reich K, Hartjen A, Reich J, Schröder J, Steingrube N, Bresch M, Ertas B, Falk TM, Blödorn-Schlicht N, Jäckle S, Meier M. Immunoglobulin E-Selective Immunoadsorption Reduces Peripheral and Skin-Bound Immunoglobulin E and Modulates Cutaneous IL-13 Expression in Severe Atopic Dermatitis. J Invest Dermatol. 2019 Mar;139(3):720-723. doi: 10.1016/j.jid.2018.09.020. Epub 2018 Oct 24. PMID: 30365931.
92. Higaki Y. [Serum IgE and Fc epsilon receptor positive lymphocytes in atopic dermatitis--relation to the background of respiratory atopy]. Nihon Hifuka Gakkai Zasshi. 1990 Mar;100(4):477-85. PMID: 2142510.
93. Ohmatsu H, Sugaya M, Suga H, Morimura S, Miyagaki T, Kai H, Kagami S, Fujita H, Asano Y, Tada Y, Kadono T, Sato S. Serum IL-31 levels are increased in patients with cutaneous T-cell lymphoma. Acta Derm Venereol. 2012 May;92(3):282-3. doi: 10.2340/00015555-1345. PMID: 22456907.
94. Kimura R, Sugita K, Yamamoto O. Successful switching to brodalumab in a patient with severe psoriasis developing ixekizumab-induced eczema. Eur J Dermatol. 2020 Dec 1;30(6):732-734. doi: 10.1684/ejd.2020.3904. PMID: 33237034.
95. Wang S, Zhu R, Gu C, Zou Y, Yin H, Xu J, Li W. Distinct clinical features and serum cytokine pattern of elderly atopic dermatitis in China. J Eur Acad Dermatol Venereol. 2020;34(10):2346-2352. doi:10.1111/jdv.16346. PMID: 32163633.
96. Wang L, Liu X, Ren M, et al. Relationship between negative emotions and skin symptoms in older patients with atopic dermatitis and related intervention strategies: a review. Geriatrics & Health Care. 2025;31(3):932-935.
97. Zhang R. Clinical characteristics and skin microbiota distribution in elderly atopic dermatitis [dissertation]. Gannan Medical University; 2025. doi:10.27959/d.cnki.ggnyx.2025.000082.
98. Chen L. Efficacy and safety of dupilumab, abrocitinib, and upadacitinib in elderly atopic dermatitis [dissertation]. Guangxi Medical University; 2025. doi:10.27038/d.cnki.ggxyu.2025.001364.
99. Chen X. Role of senescent fibroblasts in the regulation of elderly atopic dermatitis [dissertation]. Peking Union Medical College; 2025. doi:10.27648/d.cnki.gzxhu.2025.000648.
100. Zhao P, Yu C, Dou Y, et al. Clinical analysis of 108 older patients with atopic dermatitis. Journal of Clinical Dermatology. 2024;53(6):330-333. doi:10.16761/j.cnki.1000-4963.2024.06.004.
101. Fang X. Role and mechanism of natural killer cells in elderly atopic dermatitis [dissertation]. Peking Union Medical College; 2024. doi:10.27648/d.cnki.gzxhu.2024.000009.
102. Li L. Immune phenotypes of Chinese patients with atopic dermatitis and the mechanism of keratin 17-mediated mixed Th2/Th17 inflammation in elderly atopic dermatitis [dissertation]. Huazhong University of Science and Technology; 2024. doi:10.27157/d.cnki.ghzku.2024.000827.
103. Ran J. Mechanism by which fosfomycin sodium improves MC903-induced atopic dermatitis-like lesions and inflammation in mice [dissertation]. Huazhong University of Science and Technology; 2024. doi:10.27157/d.cnki.ghzku.2024.002412.
104. Jia H, Wan H, Zhang D. Changes and significance of peripheral blood Th1, Th2, and Th17 cells and squamous cell carcinoma antigen levels in older patients with atopic dermatitis. China Journal of Modern Medicine. 2023;33(19):85-91.
105. Tang Y. Clinical characteristics and related risk factors of elderly atopic-like dermatitis [dissertation]. Anhui Medical University; 2023. doi:10.26921/d.cnki.ganyu.2023.000691.
106. Hao Y. Clinical characteristics and cytokine analysis of elderly atopic dermatitis [dissertation]. Henan University; 2022. doi:10.27114/d.cnki.ghnau.2022.001587.

**2 JBI Critical Appraisal of the Six Included Studies**

Two reviewers independently assessed the methodological quality of the six studies ultimately included in the meta-analysis using the appropriate JBI critical appraisal checklists for our respective study designs.

**2.1 JBI checklist**

| **Study** | **Q1** | **Q2** | **Q3** | **Q4** | **Q5** | **Q6** | **Q7** | **Q8** | **Yes** | **No** | **Unclear** | **Overall** |
| --- | --- | --- | --- | --- | --- | --- | --- | --- | --- | --- | --- | --- |
| Zhou 2019 | Y | Y | Y | Y | Y | Y | Y | Y | 8 | 0 | 0 | Include |
| He 2020 | Y | Y | Y | Y | Y | Y | Y | Y | 8 | 0 | 0 | Include |
| Wang 2020 | Y | Y | Y | Y | Y | Y | Y | Y | 8 | 0 | 0 | Include |
| Fang 2024 | U | Y | Y | Y | Y | Y | Y | Y | 7 | 0 | 1 | Include with caveats |
| Jia 2023 | Y | Y | Y | Y | Y | N | Y | Y | 7 | 1 | 0 | Include with caveats |
| Zhao 2024 | Y | Y | Y | Y | Y | Y | Y | Y | 8 | 0 | 0 | Include |

Y = Yes; N = No; U = Unclear. Overall appraisal: Include with caveats for all six studies.

Q1	Sample inclusion criteria clearly defined

Q2	Participants and setting described in detail

Q3	Biomarker/exposure measured validly and reliably

Q4	Objective standard criteria used to define AD

Q5	Potential confounding factors identified

Q6	Strategies to address confounding stated

Q7	Disease severity/activity outcome measured validly and reliably

Q8	Statistical analysis appropriate

**2.2 Study-level evidence description**

| **Study** | **Sample(s)** | **Biomarker/sample source** | **Outcome** | **Appraisal note** |
| --- | --- | --- | --- | --- |
| Zhou 2019 | 27 | Skin CCL26 | SCORAD | SCORAD and a correlation estimate were reported. |
| He 2020 | 21 | Peripheral-blood biomarkers | SCORAD | Multiple biomarker–SCORAD correlations were available. The small older-adult subgroup and absent confounder-control information limit confidence. |
| Wang 2020 | 56 | Serum cytokines | SCORAD | SCORAD and correlation analyses were reported. |
| Fang 2024 | 9 and 7 | PBMC/blood and skin total IgE | SCORAD | Two nonoverlapping, sample-source-specific datasets were verified, but group-label inconsistencies and very small samples raise selection and precision concerns. |
| Jia 2023 | 25 | Peripheral-blood EOS and total IgE | SCCA surrogate | SCCA expression was used as a laboratory surrogate rather than a validated clinical AD severity score. |
| Zhao 2024 | 98 | Peripheral-blood biomarkers | SCORAD | This was the largest included sample and reported several biomarker–SCORAD correlations. |

Overall, the six studies included in this meta-analysis demonstrated acceptable methodological quality, though two required cautious interpretation due to specific limitations. Based on the JBI eight-item critical appraisal, four studies, Zhou 2019, He 2020, Wang 2020, and Zhao 2024, scored "Yes" on all criteria, indicating relatively high methodological quality. Fang 2024 received an "Unclear" rating for Q1 (sample inclusion criteria not clearly documented) and was therefore classified as "Include with caveats." Jia 2023 was rated "No" for Q6 (no strategies to address confounding were reported) and was also classified as "Include with caveats."

Regarding outcome assessment, five studies used SCORAD as the severity measure, whereas Jia 2023 employed SCCA expression as a laboratory surrogate rather than a validated clinical severity score. Sample sizes varied considerably across studies, ranging from 16 participants in the smallest subgroup (Fang 2024, with two non-overlapping sample-source-specific datasets of 9 and 7 participants, respectively) to 98 participants in the largest (Zhao 2024). Additionally, Fang 2024 had concerns regarding group-label inconsistencies, and He 2020 was limited by a small older-adult subgroup, both of which may reduce the robustness of the evidence to some extent.

In summary, four studies were methodologically robust, while two had specific limitations that remained within an acceptable range for inclusion. The pooled effect estimates from this meta-analysis should therefore be interpreted as exploratory associative findings derived from a small body of predominantly cross-sectional evidence, rather than as confirmatory causal inferences. These results should be evaluated in conjunction with sensitivity analyses to assess the stability of the overall findings.

3. **Discussion on PRISMA Checklist Result**

**3.1 Assessment of overall certainty of evidence**

Formal GRADE assessment was not performed because this meta-analysis synthesized correlational effect sizes (correlation coefficients r) from observational studies rather than comparative intervention effects. Instead, the overall certainty of the evidence was evaluated using a pragmatic framework comprising three complementary approaches: (1) assessment of statistical heterogeneity using the I² statistic and 95% prediction intervals; (2) methodological quality appraisal of each included study using the JBI Critical Appraisal Checklist for Analytical Cross-Sectional Studies; and (3) sensitivity analyses to examine the robustness of the pooled estimates. This approach was chosen to provide readers with a transparent, multi-dimensional basis for interpreting the strength of the evidence while acknowledging the predominantly cross-sectional and correlational nature of the available data.

**3.2 Analysis Methods and Code**

All statistical analyses were performed using R version 4.4.1 with the metafor package for meta-analyses. In addition, manual calculations using Microsoft Excel were performed to cross-check the results. The R analysis script is provided:

\# Meta-analysis of correlation coefficients

\# R version 4.4.1

\# Package: metafor

\# Effect size: Fisher's z-transformed correlation coefficient

\# Random-effects estimator: DerSimonian-Laird (DL)

\# Install metafor if it has not been installed:

\# install.packages("metafor")

library(metafor)

\# -----------------------------------------------------------------------------

\# 1. Enter the data extracted from Table 1

dat <- data.frame(

study = c("Zhao 2024", "Jia 2023", "Fang 2024 (PBMC)", "Fang 2024 (Skin)", "Zhao 2024", "Jia 2023","Zhao 2024", "Wang 2020", "Zhao 2024", "Wang 2020", "He 2020" ),

biomarker = c(rep("Total IgE", 4), rep("EOS", 2), rep("IL-4", 2), rep("IL-17", 3) ),

r = c(0.4736, 0.105, 0.733, 0.786, 0.5011, 0.607, 0.7071, 0.380, 0.7643, 0.330, 0.490),

n = c(98, 25, 9, 7, 98, 25, 98, 56, 98, 56, 21 ),

assessment = c( "SCORAD", "SCCA", "SCORAD", "SCORAD", "SCORAD", "SCCA", "SCORAD", "SCORAD", "SCORAD", "SCORAD", "SCORAD" ),

stringsAsFactors = FALSE)

\# The IL-17 subgroup combines IL-17, IL-17A, and IL-17C as one

\# biomarker family, consistent with the grouping used.

\# -----------------------------------------------------------------------------

\# 2. Convert r to Fisher's z (Table 2)

dat_es <- escalc( measure = "ZCOR", ri = r, ni = n, data = dat)

\# Equivalent manual calculations:

dat_es$Zr_manual <- atanh(dat_es$r)

dat_es$Vz_manual <- 1 / (dat_es$n - 3)

\# Display the values used in Table 2

table2_results <- dat_es[, c( "study", "biomarker", "r", "n", "assessment", "yi", "vi", "Zr_manual", "Vz_manual")]

print(table2_results)

\# -----------------------------------------------------------------------------

\# 3. Meta-analysis function ( Tables 3 and 4)

analyse_biomarker <- function(data, biomarker_name, model) {

d <- subset(data, biomarker == biomarker_name)

if (model == "Fixed-effects") { fit <- rma( yi = yi, vi = vi, data = d, method = "FE" )

} else { fit <- rma( yi = yi, vi = vi, data = d, method = "DL") }

\# Convert the pooled Fisher's z value and its 95% CI back to r

pooled <- predict(fit, transf = transf.ztor)

data.frame( Biomarker = biomarker_name, k = fit$k, Total_N = sum(d$n), Pooled_r = as.numeric(pooled$pred), CI_lower = as.numeric(pooled$ci.lb), CI_upper = as.numeric(pooled$ci.ub), Cochran_Q = fit$QE, p_heterogeneity = fit$QEp, I2_percent = fit$I2, Tau2 = fit$tau2, Effect_model = model, stringsAsFactors = FALSE)}

\# 4. Run the four biomarker-specific meta-analyses

results_raw <- rbind( analyse_biomarker(dat_es, "Total IgE", "Random-effects"), analyse_biomarker(dat_es, "EOS", "Fixed-effects"), analyse_biomarker(dat_es, "IL-4", "Random-effects"), analyse_biomarker(dat_es, "IL-17", "Random-effects"))

\# Retain the unrounded results for checking

print(results_raw)
